# Hedgehog signaling establishes precursors for germline stem cell niches by regulating cell adhesion

**DOI:** 10.1083/jcb.201610063

**Published:** 2017-05-01

**Authors:** Chun-Ming Lai, Kun-Yang Lin, Shih-Han Kao, Yi-Ning Chen, Fu Huang, Hwei-Jan Hsu

**Affiliations:** 1 Molecular and Biological Agricultural Sciences Program, Taiwan International Graduate Program, Academia Sinica and National Chung-Hsing University, Taipei 11529, Taiwan; 2 Graduate Institute of Biotechnology, National Chung-Hsing University, Taichung 40227, Taiwan; 3 Biotechnology Center, National Chung-Hsing University, Taichung 40227, Taiwan; 4 Institute of Cellular and Organismic Biology, Academia Sinica, Taipei 11529, Taiwan; 5 Institute of Molecular and Cell Biology, Academia Sinica, Taipei 11529, Taiwan; 6 Institute of Biological Chemistry, Academia Sinica, Taipei 11529, Taiwan

## Abstract

Stem cells require different types of supporting cells, or niches, to control stem cell maintenance and differentiation. However, little is known about how those niches are formed. We report that in the development of the *Drosophila melanogaster* ovary, the Hedgehog (Hh) gradient sets differential cell affinity for somatic gonadal precursors to specify stromal intermingled cells, which contributes to both germline stem cell maintenance and differentiation niches in the adult. We also report that Traffic Jam (an orthologue of a large Maf transcription factor in mammals) is a novel transcriptional target of Hh signaling to control cell–cell adhesion by negative regulation of E-cadherin expression. Our results demonstrate the role of Hh signaling in niche establishment by segregating somatic cell lineages for differentiation.

## Abstract (precis)

The mechanisms of stem cell niche formation are largely unknown. Lai et al. show that proper formation of the *Drosophila melanogaster* adult ovarian germline stem cell niche requires a Hedgehog gradient, signaling through a Ci–Traffic Jam–E-cadherin regulatory axis, to direct segregation of intermingled cells by differential cell affinity.

## Introduction

Stem cells reside in microenvironments, called niches, formed to recruit stem cells during development and regulate stem cell identify and behavior in adults (Jones and Wagers, 2008). However, despite intense research into stem cell regulation by niches, little is known about how they are specified.

**Figure 1.**
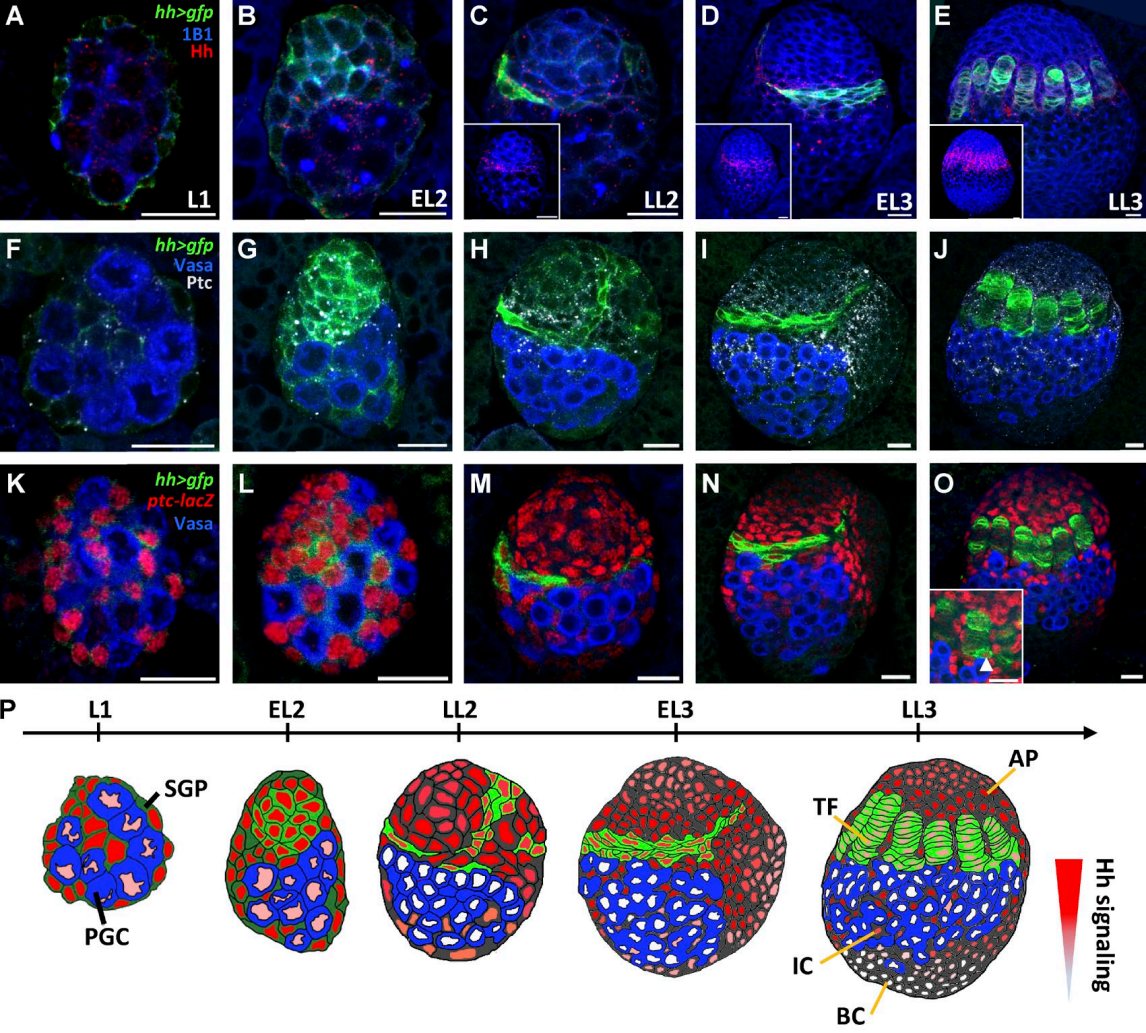
**Expression patterns of Hh, Ptc, and *ptc-lacZ* in larval ovaries.** Female gonads of L1 (A, F, and K), early L2 (EL2; B, G, and L), late-L2 (LL2; C, H, and M), early L3 (EL3; D, I, and N), and late-L3 (LL3) larvae (E, J, and O) with *hh>gfp* (green, A–O), 1B1 (blue, fusomes and somatic cell membranes, A–E), Hh (red, A–E), Vasa (blue, PGCs, F–O), Ptc (gray, F–J), and *ptc-lacZ* (red, an Hh signaling reporter, K–O). Insets in C–E show Hh distribution in gonads; inset in O shows a cap cell expressing *hh-GAL4* and *ptc-lacZ* (arrowhead). Bars, 10 µm. (P) Schematic of Hh-producing and -receiving cells of larval ovaries. The red gradient indicates strength of Hh signaling. AP, apical cell; BC, basal cell.

**Figure 2.**
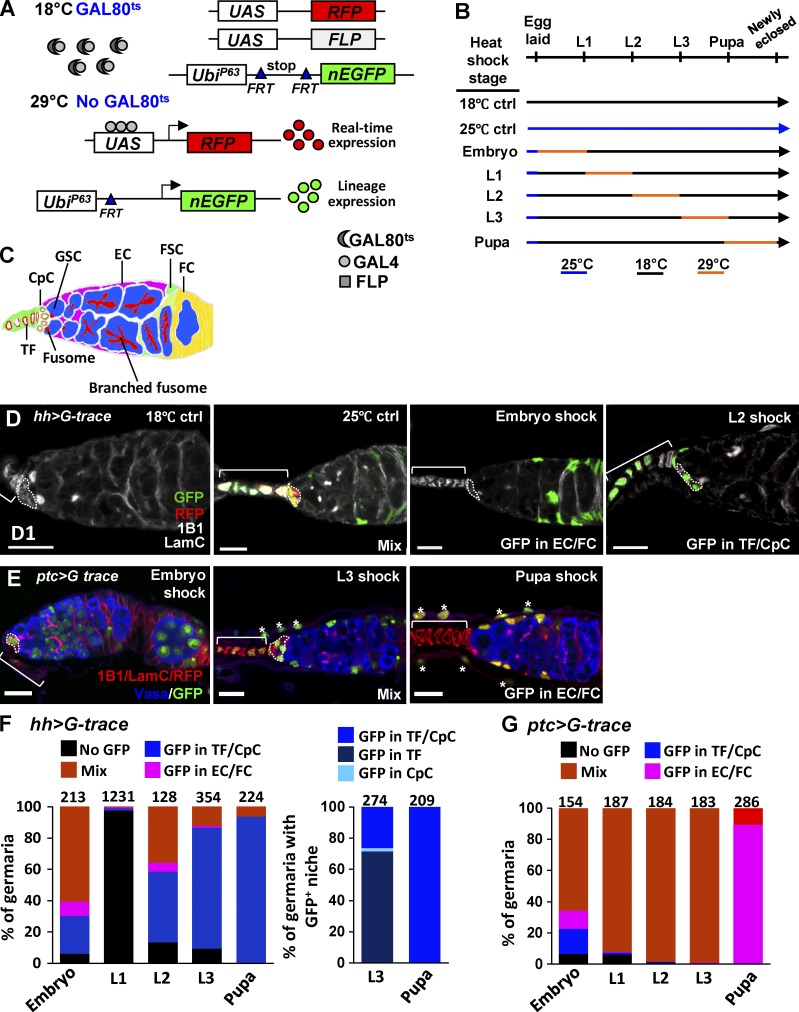
**Gonadal *hh-GAL4*- and *ptc-GAL4*-expressing cells contribute to adult GSC niches.** (A) Schematic of the G-TRACE system controlled by GAL80^ts^. At 18°C, GAL80^ts^ suppresses the activity of GAL4, driven by a specific promoter. At 29°C, GAL80^ts^ is degraded, and GAL4 activates expressions of RFP (representing real-time expression of GAL4) and Flipase (FLP). The expression of FLP in cells excises the Flipase recognition target (FRT)–flanked stop cassette separating the *Ubi-p63E* promoter and the nuclear EGFP (nEGFP) coding region to initiate GFP expression, which is maintained in all daughter cells. (B) Schematic of the strategy for tracing *hh* and *ptc-GAL4*-expressing cells during development. Embryos were collected at 25°C (blue lines) and maintained at 18°C (black lines), except at the stage of G-TRACE activation at 29°C (orange lines), and dissected 1 day after eclosion. (C) *Drosophila* germarium with germline stem cells (GSCs), terminal filaments (TFs), cap cells (CpCs), escort cells (ECs), follicle stem cells (FSCs), and follicle cells (FCs). (D) One-day (D1)–old germaria carrying *hh>G-trace* without activation (left) or activated throughout development (left middle), at the embryo (right middle) and L2 (right) stages with 1B1 (gray, fusomes) and LamC (gray, TF and cap cell nuclear envelopes). (E) D1 germaria carrying *ptc>G-trace* activated at the embryo (left), L3 (middle), and pupal (right) stages with 1B1 (red), LamC (red), and Vasa (blue). The germaria are grouped by those expressing GFP only in TFs and/or CpCs (GFP in TF/CpC, D, right), only in ECs and/or FCs (GFP in EC/FC, D, right middle, and E, right), or in both TFs and CpCs in addition to ECs and/or FCs, showing a mixed (Mix) pattern (D, left middle, and E, middle). Asterisks in E, middle and right, sheath cells. Bars, 10 µm. (F and G) Percentage of *hh>G-trace* (F) and *ptc>G-trace* germaria (G) activated at the indicated stage with no GFP, GFP in TFs/CpCs, GFP in EC/FCs, or a mixed (Mix) pattern. (F, right) Percentage of *hh>G-trace* germaria activated at the L3 or pupal stage with GFP only in TFs, in CpCs, or in both TFs and CpCs. Statistical differences in F and G were analyzed by χ^2^. Numbers of analyzed germaria are shown above each bar.

The *Drosophila melanogaster* ovary is an excellent model to address this issue, because the development of the adult ovary from the embryonic gonad involves only a small number of specific cell types, occurs progressively (Fig. 1 P; Gilboa, 2015), and contains well-characterized germline stem cells (GSCs) and niches (Wong et al., 2005). Through coalescence of primordial germ cells (PGCs; each with a unique membranous organelle, or fusome) and somatic gonadal precursors (SGPs) derived from the mesoderm (Williamson and Lehmann, 1996), the *Drosophila* ovary forms a sphere at the end of embryogenesis. During larval stages, with increased numbers of PGCs and SGPs and induced morphogenetic movements along the anterior–posterior and medial–lateral axis, the ovary forms a two-dimensional array of 16–20 stacks of somatic cells called terminal filaments (TFs; Sahut-Barnola et al., 1995). During pupariation, apical somatic cells migrate basally between TFs and through intermingled cells (ICs; which locate at the middle region of the gonad and interact with PGCs) and basal cells (which locate at the bottom of the gonad) to form 16–20 ovarioles (Cohen et al., 2002), functional units that produce eggs (Spradling, 1993). Basal cells form basal stalks that connect ovarioles to the oviduct (King et al., 1968). The anterior-most structure of the ovariole, the germarium (Fig. 2 C), houses two to three GSCs; each of their fusomes faces cap cells (a major GSC maintenance niche component), which are adjacent to basal TFs (Kirilly and Xie, 2007). GSC progeny are wrapped by escort cells (the differentiation niche) with long cellular processes and move toward the posterior of the germarium (Chen et al., 2011), where they are surrounded by a layer of follicle cells (Kirilly and Xie, 2007). The entire structure buds off from the germarium to form a new egg chamber, which develops into a mature egg. The loss of cap cells results in GSC loss (Song et al., 2007; Hsu and Drummond-Barbosa, 2009), and dysfunction of escort cells causes accumulation of undifferentiated GSCs (Jin et al., 2013; Wang et al., 2015), signifying the importance of these niches. However, how niche cap and escort cells are specified is unclear.

Hedgehog (Hh) signaling is highly conserved and controls several developmental processes (Huangfu and Anderson, 2006; Ruiz-Gómez et al., 2007; Ingham et al., 2011). Hh proteins are secreted ligands that bind to their receptor Patched (Ptc). In the absence of Hh, Ptc suppresses cell-surface localization of Smoothened (Smo), allowing Cubitus interruptus (Ci; an orthologue of Gli in mammals) to be proteolytically processed into a transcriptional repressor. On binding of Hh to Ptc, Ptc inhibition of Smo is relieved and Smo accumulates at the plasma membrane; thus, full-length Ci is released and acts as a transcriptional activator. *Drosophila* has one homologue for each component of the Hh signaling pathway (Ingham et al., 2011). *hh* transcripts and Hh proteins are expressed in TFs of late-L3 female larval gonads (Forbes et al., 1996; Gancz et al., 2011), but whether Hh signaling is involved in niche establishment is unexplored.

We show that Hh signaling specifies stromal ICs, which contribute to adult GSC niches. SGPs located posteriorly to TFs receive Hh signals to suppress E-cadherin expression, thus becoming ICs to intermingle with PGCs. Hh signaling–defective ICs exhibit a high level of E-cadherin expression, displaying epithelial basal cell characteristics, and do not intermingle with PGCs; thus, in such gonads, PGCs aggregate and form a large cluster. The loss of ICs further results in the reduction of adult niche cap and escort cells and GSCs. Conversely, hyperactivation of Hh signaling in SGPs forms ectopic ICs accompanied by increased PGCs and the absence of basal cells, resulting in the expansion of escort cells. We also report that Hh signaling maintains IC cell fate by Traffic Jam (Tj)–mediated suppression of E-cadherin expression. Clonal analysis reveals that *smo*-defective ICs exhibit increased E-cadherin expression but decreased Tj expression; in contrast, overexpression of *tj* in ICs suppresses E-cadherin expression. Decreased E-cadherin expression or enhanced *tj* expression in *smo*-defective ICs partially rescues the IC pool and soma-germline interaction defect. Furthermore, *tj*- or *shotgun* (*shg*; encoding E-cadherin)–knockdown cells are excluded from the IC region. These results suggest that Hh signaling controls the segregation of ICs and basal cells via control of cell-cell adhesion, an effect that is mediated by Tj–E-cadherin regulation. Finally, we report that Tj is a novel target of Ci in response to Hh signals. Collectively, our results show that Hh signaling controls the formation of GSC niche precursors through its developmental role in promoting IC fate by which Ci directly targets Tj. These studies add insight into how an organ uses differential cell affinity to generate niches.

## Results

### Hh proteins produced from TFs signal to apical somatic cells and ICs

To examine if Hh signaling is involved in niche formation, we first characterized Hh-producing and Hh-receiving cells in larval gonads. We used *UAS-gfp* driven by *hh-GAL4* (Sarikaya and Extavour, 2015) to mark *hh*-expressing cells. *hh-GAL4* was expressed in SGPs by the early L2 stage (Fig. 1, A, B, F, G, K, and L), and its expression was restricted to disc-shaped cells, forming a crown-like structure in the anterior portion of late-L2 and early-L3 larval gonads (Fig. 1, C, D, H, I, M, and N). In the late-L3 gonad, 7 to 10 disc-shaped cells formed a TF where *hh-GAL4* was continuously expressed (Fig. 1, E, J, and O). Unlike *hh-GAL4* expression, Hh expression showed punctate patterns in both SGPs and PGCs by the early-L2 stage (Fig. 1, A and B), whereas it was strongly expressed in TFs and gradually reduced in cells adjacent to TFs after the late-L2 stage (Fig. 1, C–E). We identified Hh signal–receiving cells by examining expression of Ptc, and *ptc-lacZ*, an Hh signaling reporter (Chen and Struhl, 1996). Ptc (expressed as punctate granules) and *ptc-lacZ* were expressed primarily in SGPs but also in PGCs of L1 and L2 gonads (Fig. 1, F, G, K, and L). In L3 gonads, Ptc and *ptc-lacZ* were excluded from TFs but strongly present in cells adjacent to TFs, including ICs (Fig. 1, H–J and M–O). Our results suggest that Hh acts as an autocrine signal in SGPs of early larval gonads, whereas in late larval gonads, Hh is mainly produced by TFs and signals to apical somatic cells and ICs (Fig. 1 P).

### Gonadal Hh signaling–producing cells contribute to adult GSC maintenance niches

To identify cell types that are derived from gonadal Hh-producing and Hh-receiving cells, we used the GAL4 technique for real-time and clonal expression (G-TRACE) system to trace gonadal cells that expressed *hh-GAL4* and *ptc-GAL4*, which mimic the expression of Hh (see Fig. 1) and Ptc (Fig. S1), respectively. G-TRACE combined GAL4/UAS, Flipase (FLP)/FLP recognition target (FRT), and fluorescent reporters to report real-time expression of *GAL4* (*UAS-RFP*) and permanent labeling (*ubi-GFP*) of cells that were expressing *GAL4* (Fig. 2 A; Evans et al., 2009). We also used a temperature-sensitive mutant of GAL80 (GAL80^ts^) to control activation of the G-TRACE cassette at the indicated stage (Fig. 2 B). *hh>G-trace* flies cultured at a permissive temperature, 18°C, throughout development showed no red fluorescent protein (RFP) or GFP expression in 1-d-old germaria (Fig. 2, C and D). At a nonpermissive temperature (25°C), RFP was expressed in TF and cap cells (maintenance niches), and GFP was present in TF, cap, escort (differentiation niche), and follicle cells, displaying a mixed pattern (Fig. 2, D, left middle). In addition, after activation at each stage, RFP expression mimicked *hh-GAL4* expression (see Fig. 1), and GFP was well expressed in *hh>G-trace* gonads (Fig. S1, A–D). These results demonstrated the availability of the G-TRACE cassette, which was precisely controlled by GAL4 together with GAL80^ts^. G-TRACE driven by *hh-GAL4* during embryogenesis resulted in 61% of germaria (*n* = 213) showing as GFP positive in a mixed pattern, but without RFP expression (because larvae were kept at 18°C after embryogenesis; Fig. 2, D [right middle] and F), suggesting the multipotency of embryonic SGPs. Of the germaria, 97% (*n* = 1,231) had no GFP expression when G-TRACE was activated at the L1 stage, reflecting the low expression of *hh-GAL4* (see Figs. 1 F and S1 B). Notably, the proportion of germaria that expressed GFP in maintenance niches, induced at the L2 and pupal stages, increased from 45% (*n* = 128) to 94% (*n* = 224; Fig. 2, D [right middle] and F). A similar observation was found in late-L3 *hh>G-trace* gonads, in which GFP-positive cells marked after the L1 stage were more restricted in TFs (Fig. S1, E–H). We found that 72% of germaria (*n* = 274) with GFP expression in maintenance niches induced at the L3 stage (Fig. 2 F, right) carried GFP only in TFs, whereas 27% carried GFP in both TF and cap cells. However, all germaria (*n* = 209) expressed GFP in both TF and cap cells when GFP was induced at the pupal stage (Fig. 2 F, right). These results all indicate that Hh-producing cells contribute to adult niche cap cells.

### Hh signaling–receiving cells contribute to both GSC maintenance and differentiation niches

G-TRACE activated by *ptc-GAL4* during embryogenesis induced GFP expression in germ cells in 27% of germaria (*n* = 154; Fig. 2 E) and in a mixed pattern in 66% of germaria (*n* = 154; Fig. 2 G). After the embryonic stage, GFP expression induced at L1 and L2 stages exhibited a mixed pattern in most late-L3 gonads (Fig. S1, J and K) and 1-d-old germaria (Fig. 2 G). Cells marked GFP at the L3 stage were mostly excluded from TFs in the late-L3 gonad (Fig. S1 L) but exhibited a mixed pattern in D1 germaria (Fig. 2, middle). Further, 89% of germaria (*n* = 286) expressed GFP only in escort and follicle cells when G-TRACE was activated during pupation (Fig. 2, E [right] and G). Apical cells and basal cells are not incorporated into germaria (King et al., 1968; Cohen et al., 2002), suggesting that *ptc-GAL4*–expressing cells (ICs) are the source for niche cap and escort cells. We confirmed this by activating *G-trace* using *tj-GAL4*, which was expressed in ICs and basal cells at the late-L3 stage (Fig. S1, M–R). Notably, both *hh-GAL4–* and *ptc-GAL4*–expressing cells were able to contribute to niche cap cells, an observation supported by the results that forming niche cap cells expressed both Hh and *ptc-lacZ* (see Fig. 1 O). From these results, we conclude that ICs receive Hh signals and contribute to niche cap and escort cells.

### Somatic Hh signaling maintains the IC pool for niche formation

**Figure 3.**
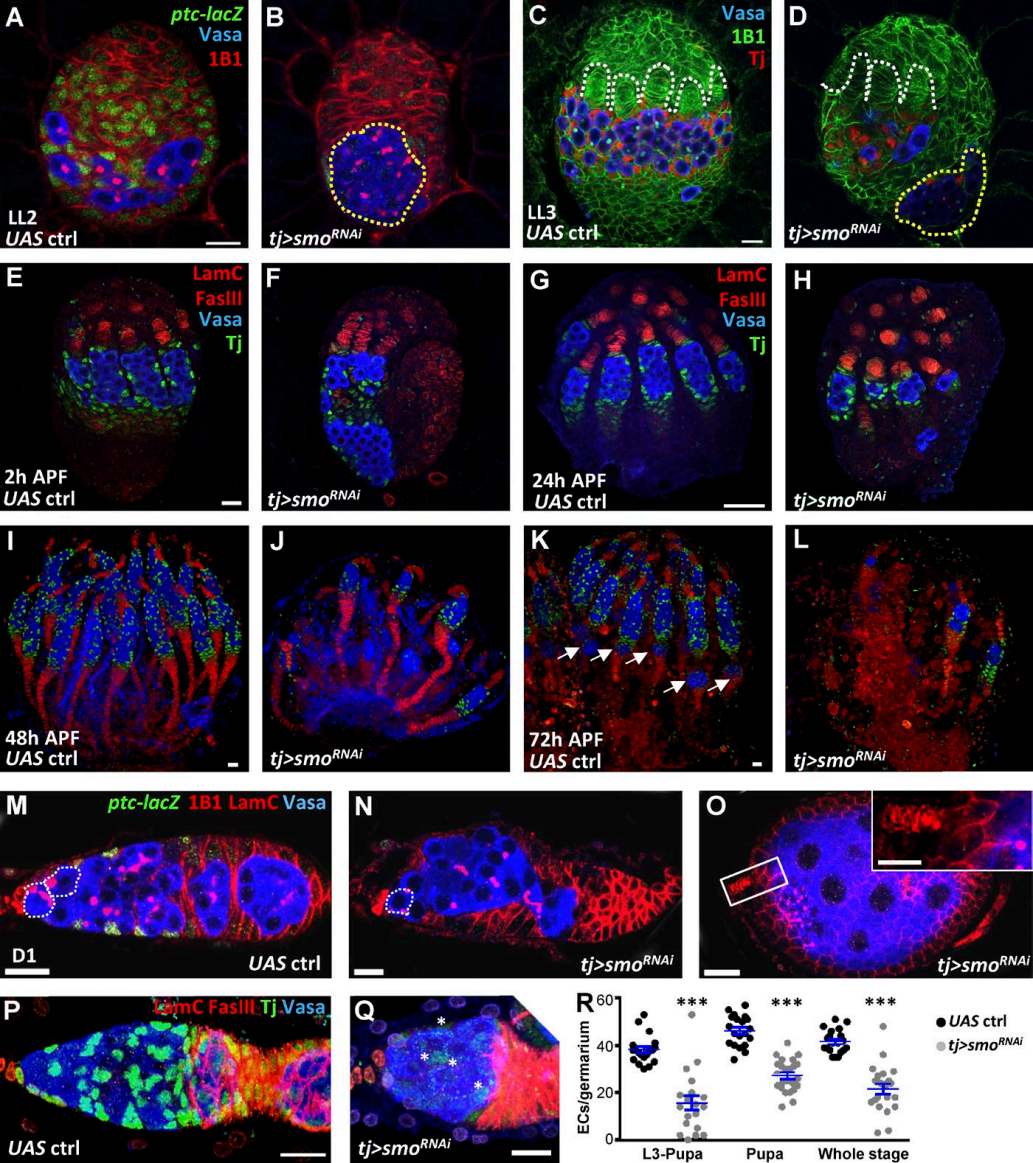
**Somatic Hh signaling controls soma**–**germline interaction and GSC niche formation.** (A–D) Control (ctrl; A and C) and *tj>smo^RNAi^* gonads (B and D) of late-L2 (A and B) and late-L3 (C and D) larvae with Vasa (blue, PGCs), 1B1 (red in A and B, green in C and D, fusomes and somatic cell membranes), *ptc-lacZ* in A and B (green, an Hh signaling reporter), and Tj in C and D (red, ICs). White dashed lines, TFs; yellow dashed circles, PGC clusters. (E–L) Control (E, G, I, and K) and *tj>smo^RNAi^* pupal ovaries (F, H, J, and L) with Vasa (blue), LamC (red, TF and cap cell nuclear envelopes), FasIII (red, stalk cell membranes), and Tj (green) at 2 (E and F), 24 (G and H), 48 (I and J), and 72 h (K and L) after puparium formation (APF). Arrows in K, newly formed egg chambers. (M–Q) One-day (D1) –old control (M and P) and *tj>smo^RNAi^* ovaries (N, O, and Q) with *ptc-lacZ* in M–O (green), Vasa (blue, germ cells), 1B1 in M–O (red, fusomes and follicle cell membranes), LamC (red), FasIII in P and Q (red, follicle cell membranes), and Tj expression in P and Q (green, cap, escort and follicle cell nuclei). The inset in O shows an empty germarium; asterisks in Q show escort cells with weak Tj expression. Dashed circles, GSCs. Bars, 10 µm. (R) Escort cell (EC) number per germarium of D1 control and flies with *smo*-knockdown driven by *tj-GAL4* from L3 to pupal stages, during the pupal stage, or throughout development (whole stage). Statistical differences were analyzed by two-tailed *t*-test. Error bars represent SEM. ***, P < 0.001.

To investigate the role of Hh signaling in ICs, we used *tj-GAL4* to drive a *smo^RNAi^* line (Bloomington) in ovaries at different developmental stages. In the late-L2 control gonad, *ptc-lacZ* was expressed in apical cells and ICs that were well intermingled with PGCs (Fig. 3 A), whereas *ptc-lacZ* expression was nearly abolished and PGCs clustered together in the *tj>smo^RNAi^* gonad (Fig. 3 B), showing the efficiency of the *RNAi* line. In the late-L3 control gonad, TFs were present and PGCs intermingled with ICs that expressed Tj (Fig. 3 C), whereas a large PGC cluster was located at the bottom of *tj*>*smo^RNAi^* gonads and a few PGCs interacted with ICs closer to TFs (Fig. 3 D and Fig. S2, A and B). Similar phenomena were observed when other SGP drivers, *ptc-GAL4* or *bab1-GAL4* (Bolívar et al., 2006), drove the same *smo^RNAi^* line or when *tj-GAL4* drove another *UAS-smo^RNAi^* (NIG; Fig. S2, C–H). Significantly, *tj*>*smo^RNAi^* gonads carried fewer ICs (485 ± 194, *n* = 9) than did controls (716 ± 114, *n* = 7). MA33, another IC marker (Gilboa and Lehmann, 2006), was also dramatically reduced in ICs; instead, cells in the IC region expressed a level of F-actin similar to that of basal cells (Fig. S2, I–L). These results suggest that Hh signaling maintains the IC pool.

At 2 h after puparium formation (APF), germaria were formed and ICs incorporated into each germarium; these ICs were identified as escort cells (Fig. 3, E and G), and at 48 h APF, germaria were completely formed with basal stalks, derived from basal cells, marked by Fasciclin III (FasIII; Fig. 3 I). At 72 h APF, a newly formed egg chamber was located posterior to the germarium (Fig. 3 K, arrows), indicating the onset of oogenesis. However, in the *tj*>*smo^RNAi^* ovary at 2 h APF, a large PGC cluster was still present, and only a few ICs intermingled with PGCs located closer to TFs (Fig. 3 F). Germaria carried fewer PGCs and escort cells, and each of them connected to a basal stalk in *smo*-knockdown ovaries (Fig. 3, H, J, and L). The PGC cluster was absent in the *tj*>*smo^RNAi^* ovary at 24 h APF. Germaria without PGCs likely undergo degeneration, thus forming fewer ovarioles in the adult. Indeed, 1-d-old *tj*>*smo^RNAi^* ovaries (*n* = 24) carried only 6.9 ± 2.4 ovarioles in comparison with 17.7 ± 1.7 ovarioles in control ovaries (*tj>mCD8-gfp*, *n* = 22, P < 0.001).

As a consequence, 1-d-old *tj>smo^RNAi^* germaria with the absence of *ptc-lacZ* had a severe reduction in GSCs and niche cap cells (Fig. 3, M–O; and Fig. S3 A), and some were empty with a large egg chamber (Fig. 3 O). In contrast to control germaria (42 ± 5, *n* = 20) germaria with *smo* knockdown throughout development carried fewer escort cells (26 ± 10, *n* = 21, P < 0.001; Fig. 3, P–R). A similar degree of escort cell reduction was observed in gonads with *smo* knockdown from L3 to pupal stages, whereas suppression of *smo* expression only during pupation caused less reduction of escort cells (Fig. 3 R), suggesting that Hh signaling is important at this period for there to be escort cells in the adult. The anterior-most PGCs receive bone morphogenetic protein signals from maintenance niches to adapt GSC fate (Song et al., 2004). Bone morphogenetic protein signaling in GSCs, as revealed by p-Mad expression and the number of p-Mad–positive GSCs, was reduced in late-L3 *tj>smo^RNAi^* gonads, as compared with controls (Fig. S3, B–E), indicating impairment of GSC recruitment. Our results clearly show that Hh signaling maintains ICs for niche formation, as well as GSC recruitment.

### Forcing Hh signaling expands the IC population

**Figure 4.**
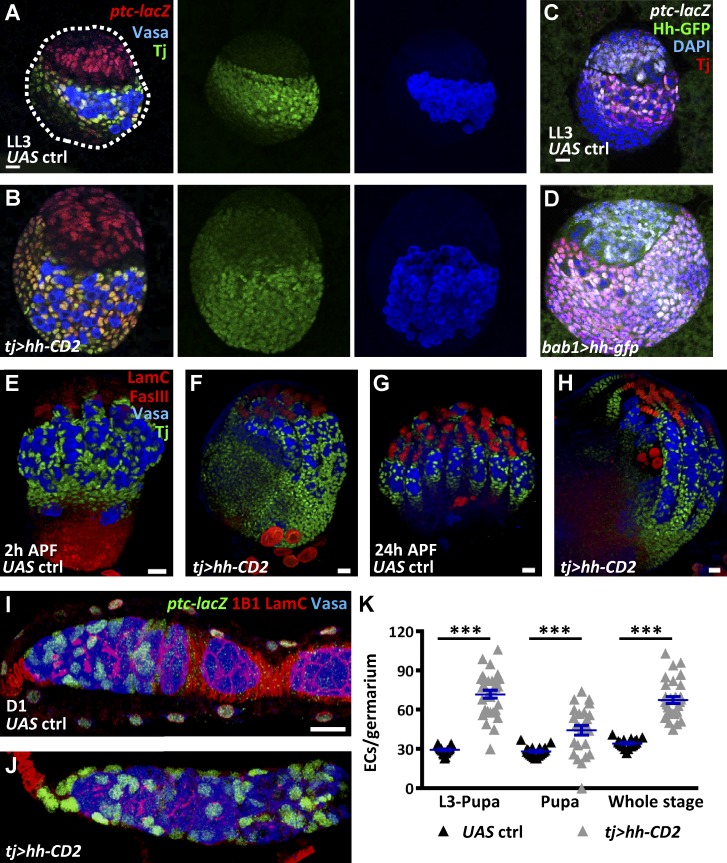
**Overexpression of Hh promotes IC formation leading to expansion of escort cells.** (A and B) Late-L3 (LL3) control (ctrl; A) and *tj>hh-CD2* gonads (B) with Vasa (blue, PGCs), *ptc-lacZ* (red, an Hh signaling reporter), and Tj (green, ICs). Dashed line in A outlines the gonad. (C and D) LL3 control (ctrl; C) and *bab1>hh-gfp* gonads (D) with GFP (green), *ptc-lacZ* (gray), Tj (red), and DAPI (blue, DNA). (E–H) Control (E and G) and *tj>hh-CD2* pupal ovaries (F and H) with Vasa (blue), LamC (red, TF and cap cell nuclear envelopes), FasIII (red, stalk cell membranes), and Tj (green) at 2 (E and F) and 24 h (G and H) after puparium formation (APF). (I and J) 1-d-old (D1) control (I) and *tj>hh-CD2* ovaries (J) with *ptc-lacZ* (green, escort cells), Vasa (blue, germ cells), 1B1 (red, fusomes and follicle cell membranes), and LamC (red). Bars, 10 µm. (K) Escort cell (EC) number per germarium of D1 control and flies with *hh-CD2* overexpression driven by *tj-GAL4* from L3 to pupal stages, during the pupal stage, or throughout development (whole stage). Statistical differences were analyzed by two-tailed *t*-test. Error bars represent SEM. ***, P < 0.001.

Conversely, hyperactivation of Hh signaling in SGPs caused enlarged gonads accompanied by expanded ICs and PGCs at the late-L3 stage (Fig. 4, A–D) when *tj-GAL4* and *bab1-GAL4* were used to drive *UAS-hh-CD2* (membrane-bound Hh) and *UAS-hh-gfp* (diffusible Hh; Tanimoto et al., 2000; Torroja et al., 2004), respectively. In control gonads, ICs (Tj-positive cells) intermingled with PGCs and occupied the middle portion of the gonad, whereas basal cells did not express Tj and remained separate from PGCs. In contrast, in *hh*-overexpressing gonads, nearly every somatic cell, except apical and TF cells, expressed both *ptc-lacZ* and Tj and were well intermingled with PGCs. In addition, we did not detect *ptc-lacZ* expression in PGCs of *tj>hh-CD2* or *tj>hh-gfp* gonads, suggesting that the expansion of PGCs resulted from hyperactivation of Hh signaling in ICs. These results indicate that Hh signaling has the capacity to direct SGPs to become ICs and suppresses the formation of basal cells.

As expected, the expanded ICs in *hh*-overexpressing ovaries resulted in ectopic escort cells in germaria (Fig. 4, E–K). Forcing *hh* expression in gonads from L3 to pupal stages, or throughout development, showed a comparable increase in escort cell numbers (Fig. 4 K), suggesting that somatic cells posterior to TFs are sensitive to Hh signaling for cell fate choice at the L3 to pupal stage. Similar results were obtained using another escort cell marker, *PZ1444* (Margolis and Spradling, 1995; Kirilly et al., 2011; Fig. S3, F–H). Surprisingly, we did not observe an increased number of niche cap cells or GSCs in *hh*-overexpressing germaria (Fig. S3 I). Instead, escort cells were often found in the space where niche cap cells were originally located (Fig. 4 J). Combining the data showing ICs of *tj>smo^RNAi^* with decreased IC markers but increased basal cell markers (Fig. S2, I–L), we hypothesized that Hh signaling promotes ICs, which contribute GSC niches, but suppresses basal cells, which form basal stalks.

### Hh signaling controls the cell fate decision between stromal ICs and epithelial basal cells

**Figure 5.**
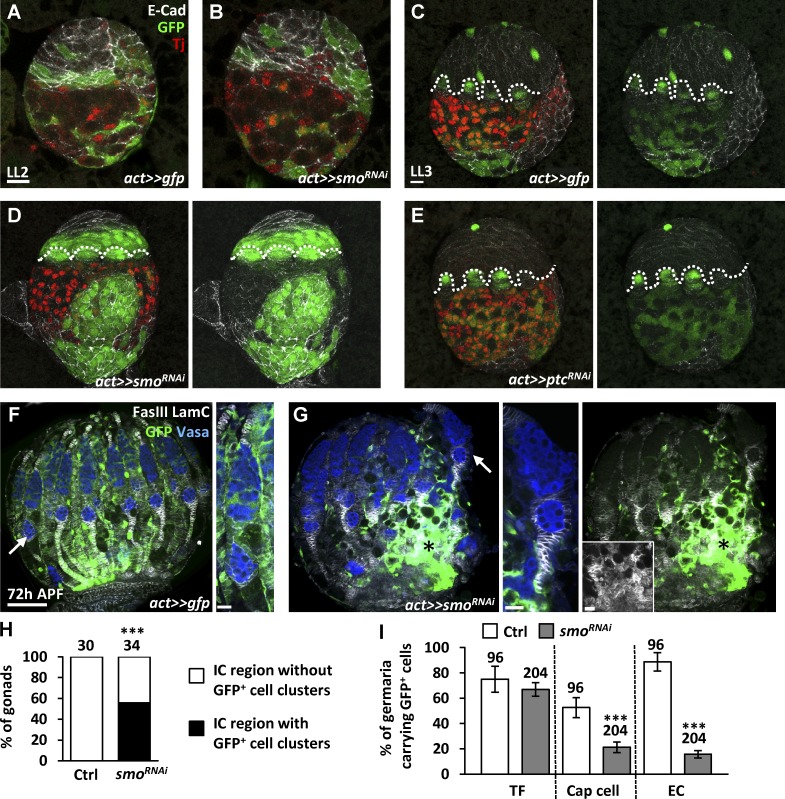
**Somatic Hh signaling controls cell fate decision between stromal ICs and epithelial basal cells.** (A–E) Control (*act>>gfp*; A and C), *smo-* (B and D), and *ptc-*knockdown (E) mosaic gonads with GFP (green, flip-out clones), E-cad (gray), and Tj expression (red, ICs) of late-L2 (A and B) and late-L3 larvae (C–E). Dashed lines mark forming TFs. (F and G) Mock control (F) and *smo-*knockdown mosaic pupal ovaries (G) with GFP (green, flip-out clones), Vasa (blue, germ cells), LamC (gray, TF and cap cell nuclear envelopes), and FasIII (gray, stalk cell membranes) 72 h after puparium formation (APF). F, right, and G, middle, are an enlarged view of the germarium in (F) and (G, arrows). The inset in G, right, shows only the irregular arrangement of GFP^+^ stalk cells from the region indicated by asterisks in G, left, and G, right. Bars: (A–E; F, right; G, middle; and G, right, inset) 10 µm; (F, left, and G, left) 50 µm. (H) Percentage of gonads with or without GFP^+^ cell clusters in the IC region of late-L3 mock control and *smo*-knockdown mosaic gonads. (I) Percentage of germaria carrying GFP^+^ TFs, cap cells, and escort cells (ECs) in control and *smo*-knockdown mosaic pupal ovaries at 72 h APF. Gonad or germaria numbers analyzed are shown above each bar. ***, P < 0.001. Statistical differences analyzed by χ^2^ in H and two-tailed *t*-test in in I. Error bars represent SD in I.

To test this hypothesis, we examined the cell property of *smo*-knockdown cells by examining the expression of Tj, an IC marker, which suppresses E-cadherin expression in follicle cells (Li et al., 2003). We generated *smo*-knockdown SGPs in early-L1 larvae by using a 10-min heat shock to activate flip-out GAL4 (*actin promoter-FRT-CD2-FRT-GAL4*) and traced them at different developmental stages (Fig. 5). *smo^RNAi^* was expressed when the stop codon flanked by two FRT sites was excised by FLP (*smo*-knockdown cells were characterized by the presence of GFP). We did not observe dramatic differences between control and *smo*-knockdown cells in late-L2 gonads (Fig. 5, A and B); at this stage, basal cells were not formed. However, late-L3 *smo-*knockdown cells were gathered in the IC region of 56% of mosaic gonads (*n* = 34) in comparison with control gonads (*n* = 30, P < 0.001; Fig. 5, C, D, and H). These *smo*-knockdown cells did not express Tj but expressed levels of E-cadherin comparable to that in basal cells (Fig. 5, C and D), suggesting that *smo*-knockdown cells exhibit basal cell characteristics. In contrast, *ptc*-knockdown cells (forced Hh signaling) generated at the early L1 stage were not only in the IC region but also in the basal region of the mosaic gonads (Fig. 5 E); these cells expressed Tj and E-cadherin at a level comparable to that in ICs and were well intermingled with PGCs (Fig. 5 E), which represents IC properties.

If *smo*-knockdown cells in the IC region tend to adopt basal cell fate, those cells should mostly contribute to stalk cells (derived from basal cells), but not cap or escort cells. Indeed, *smo*-knockdown cells generated at the early-L1 stage were not favored to contribute to cap or escort cells, examined at 72 h APF (Fig. 5, F, G, and I). Many *smo*-knockdown cells expressed FasIII, a stalk cell marker, and accumulated at the bottom of the ovary (asterisk in Fig. 5 G). Furthermore, in *tj>smo^RNAi^* gonads, we did not detect enhanced cell death or decreased epidermal growth factor receptor (Egfr) signaling (Fig. S4, A–D), which is activated by Spitz produced from PGCs to maintain IC survival (Gilboa and Lehmann, 2006). Perhaps ICs closer to PGCs in *tj>smo^RNAi^* gonads were supported by Egfr signaling. These results further support our hypothesis that Hh signaling determines IC fate.

### Hh signaling cell-autonomously controls expression of E-cadherin and Tj in ICs

**Figure 6.**
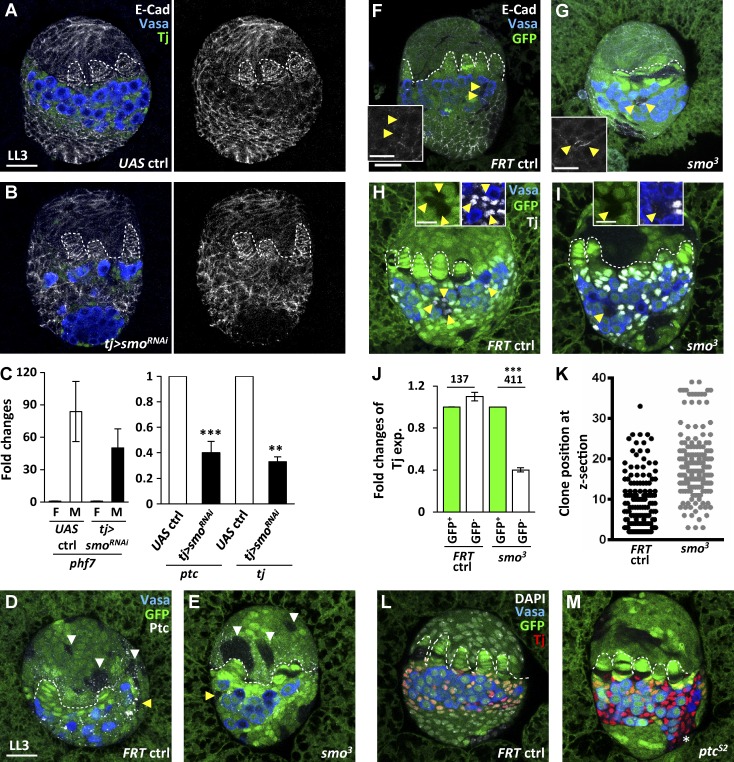
**Somatic Hh signaling directly controls Tj and E-cadherin expression in ICs.** (A and B) Late-L3 (LL3) control (ctrl; A) and *tj>smo^RNAi^* gonads (B) with E-cad (gray), Vasa (blue, germ cells), and Tj (green, ICs). (C, left) The mean fold changes of *phf7* transcripts in male (M) gonads relative to those in female (F) gonads of control and *tj>smo^RNAi^* flies. (C, right) The mean fold changes of *ptc* and *tj* transcripts in *tj>smo^RNAi^* gonads relative to those in control gonads. (D–I) LL3 mock control (D, F, and H) and *smo^3^* mutant mosaic gonads (E, G, and I) with GFP (green, wild-type cells), Vasa (blue), Ptc in D and E (gray), E-cad in F and G (gray), and Tj in H and I (gray). (J) The mean fold changes of Tj protein expression (exp.) in GFP^−^ ICs relative to those of neighboring GFP^+^ wild-type ICs of LL3 mock and *smo^3^* mutant mosaic gonads. Numbers of ICs analyzed are shown above each bar. (K) Clonal position in z-sections of control and *smo^3^* mutant mosaic gonads from the site facing the midline toward the site facing the fat body (0–40 sections). (L and M) LL3 mock control (L) and *ptc^S2^* mutant mosaic gonads (M) with GFP (green, wild-type cells), Vasa (blue), Tj (red), and DAPI (gray, DNA). The asterisk in M indicates the *ptc^S2^* mutant clones located at the bottom region but expressing Tj. Dashed lines mark forming TFs. White and yellow arrowheads indicate clones present in the apical and IC regions, respectively. Bars: (insets) 10 µm; (A, D, and F) 20 µm. *, P < 0.05; **, P < 0.01; ***, P < 0.001. Statistical differences in C and J were analyzed by two-tailed *t*-test. Error bars represent SD in C and SEM in J.

We next asked whether Hh signaling controls Tj expression in ICs. Consistently, *tj>smo^RNAi^* gonads carried fewer ICs that weakly expressed Tj and were associated with PGCs, whereas other somatic cells in the IC region did not express Tj but expressed high levels of E-cadherin characteristic of basal cells (Fig. 6, A and B). In addition, quantitative real-time RT-PCR results showed that late-L3 *tj>smo^RNAi^* female gonads expressed very low levels of the male marker *phf^7^* (Yang et al., 2012) and decreased *ptc* (a direct target of Hh signaling) and *tj* transcripts compared with controls (Fig. 6 C). These results suggest that Hh signaling controls *tj* expression at a transcriptional level. Furthermore, *smo^3^* mutant somatic cells (identified by the absence of GFP, generated by FRT-mediated mitotic recombination) exhibited decreased Ptc expression (Fig. 6, D and E), indicating impairment of Hh signaling. Expression of Tj in *smo^3^* mutant ICs was reduced to 40% of that in neighboring normal GFP-positive ICs, whereas E-cadherin expression was increased (Fig. 6, F–J), indicating that Hh signaling directly controls Tj and E-cadherin expression levels in ICs.

*smo^3^* mutant ICs, compared with ICs in controls, were located closer to the lateral aspect of gonads, facing the fat body (high number in the z-section; Fig. 6 K), where TFs are formed later than in the medial side of the gonad facing the midline of the larva (Sahut-Barnola et al., 1995). This result suggests that *smo^3^* mutant ICs may be pushed away from the forming germaria of the mosaic gonad, probably because the affinity of such cells is closer to that of cells receiving fewer Hh signals. In addition, mitotic recombination-induced *ptc^S2^* mutant ICs expanded to the basal region and expressed a high level of Tj (Fig. 6, L and M), consistent with results from the flip-out clonal assay. Tj has also been reported to promote expression of Piwi, which interacts with Piwi-interacting RNA to induce gene silencing, including FasIII (Saito et al., 2009); however, FasIII was not detectable in the control ovary until the pupal stage (Figs. 3 and 4), and Piwi was not reduced in *smo*-knockdown or mutant cells (Fig. S4, E–K), probably because the remaining amount of Tj is enough to maintain Piwi expression. Our results indicate that Hh signaling cell-autonomously controls Tj expression in ICs, and this regulation is critical to maintain proper cell affinity of ICs.

### Tj controls SGP adhesion to adopt IC fate

**Figure 7.**
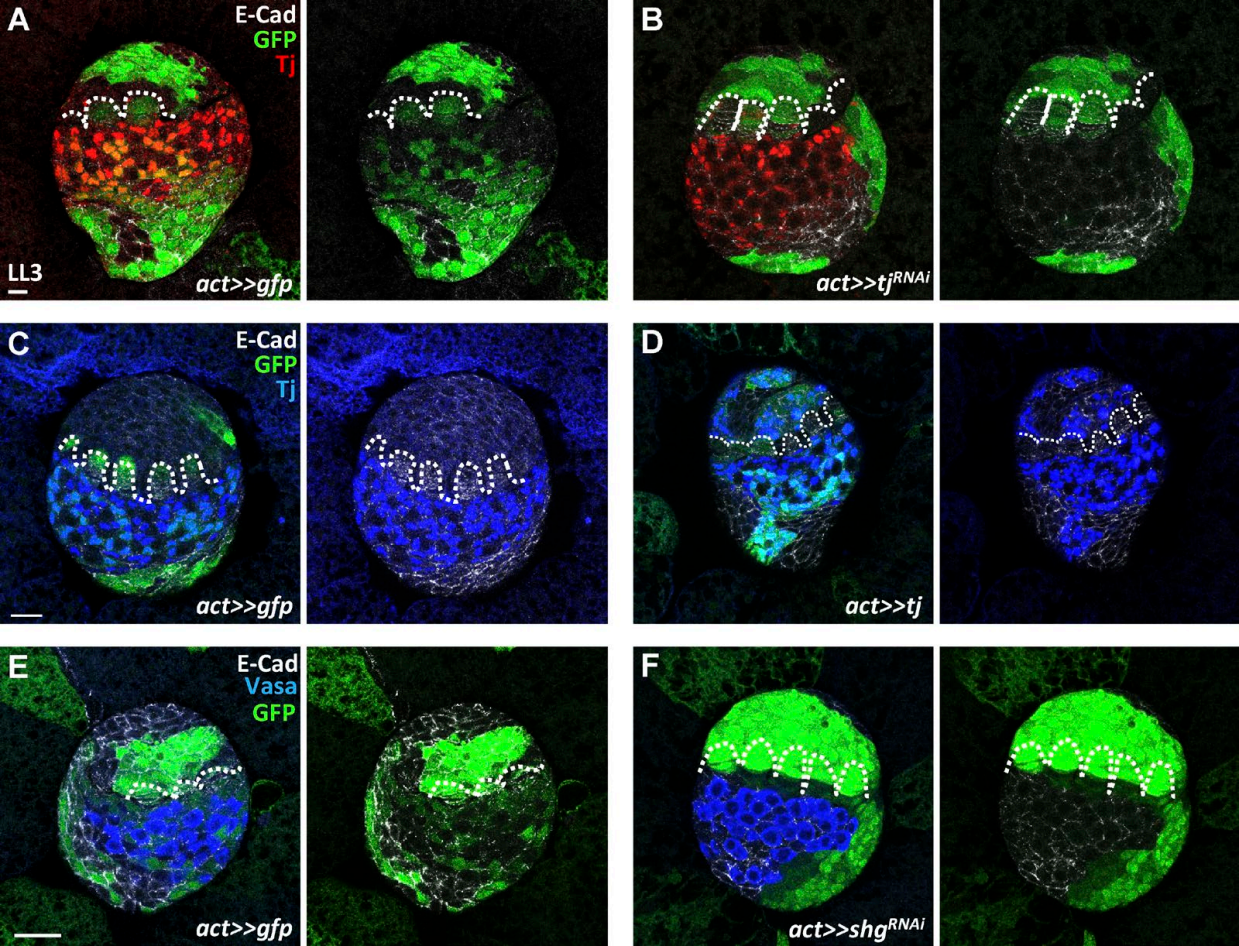
**Tj modulates E-cadherin expression for somatic cells to intermingle with PGCs and adopt IC cell fate.** (A–F) Late-L3 mock control (ctrl; A, C, and E), *tj-*knockdown (B), *tj*-overexpressing (D), and *shg-*knockdown (F) mosaic gonads with GFP (green, flip-out clones), E-cad (gray), Tj in A–D (red in A and B, blue in C and D, ICs), and Vasa in E and F (blue, PGCs). *tj^RNAi^* and *shg^RNAi^* flip-out cells do not intermingle with PGCs and only locate in apical and basal regions of mosaic gonads. *tj-*overexpressing flip-out clones exhibit low expression of E-cadherin. Dashed lines mark forming TFs. Bars: (A and C) 10 µm; (E) 20 µm.

During embryogenesis or organogenesis, cells are segregated into distinct domains and then further differentiate into specific cell lineages (Krens and Heisenberg, 2011). We thus proposed that SGPs posterior to TFs might be segregated into IC and basal regions according to their cell affinity, at least in part through the regulation of Tj-mediated suppression of E-cadherin expression. To test this possibility, we generated *tj*-knockdown or *tj*-overexpressing somatic cells (GFP positive) in early-L1 larvae by flip-out *GAL4* and traced them at the late-L3 stage (Fig. 7, A–D). In control gonads (*n* = 12; Fig. 7 A), GFP-positive control cells randomly distributed in apical, basal, and IC regions. However, most of the *tj*-knockdown somatic cells did not interact with PGCs and were localized to apical and basal regions of mosaic gonads (Fig. 7 B), where they expressed high levels of E-cadherin. Only 8% and 12% of *act>> tj^RNAi^ (II)* (*n* = 32) and *act>> tj^RNAi^ (III)* gonads (*n* = 37), respectively, carried *tj*-knockdown somatic cells in the IC region. *tj*-knockdown mosaic gonads did not exhibit reduced gonad size or carry condensed DNA accumulation (unpublished data), suggesting that *tj*-knockdown cells were unable to stay in the IC region, rather than suggesting cell death. In contrast, *tj-*overexpressing flip-out cells of late-L3 gonads formed a cluster with decreased E-cadherin expression (Fig. 7 C, and D), consistent with our previous finding that Tj negatively controls E-cadherin expression in ICs. Decreased E-cadherin expression in somatic cells in L1 larvae with *flip-out GAL4* to drive RNAi against *shg* also caused cells to adhere together outside the IC region in the late-L3 stage (*n* = 18; Fig. 7, E and F). These results suggest that a proper level of E-cadherin expression mediated by Tj is required for SGPs to become ICs.

### Decreasing *shg* or overexpressing *tj* in *smo-*knockdown somatic cells rescues soma–germline interaction and the IC population

**Figure 8.**
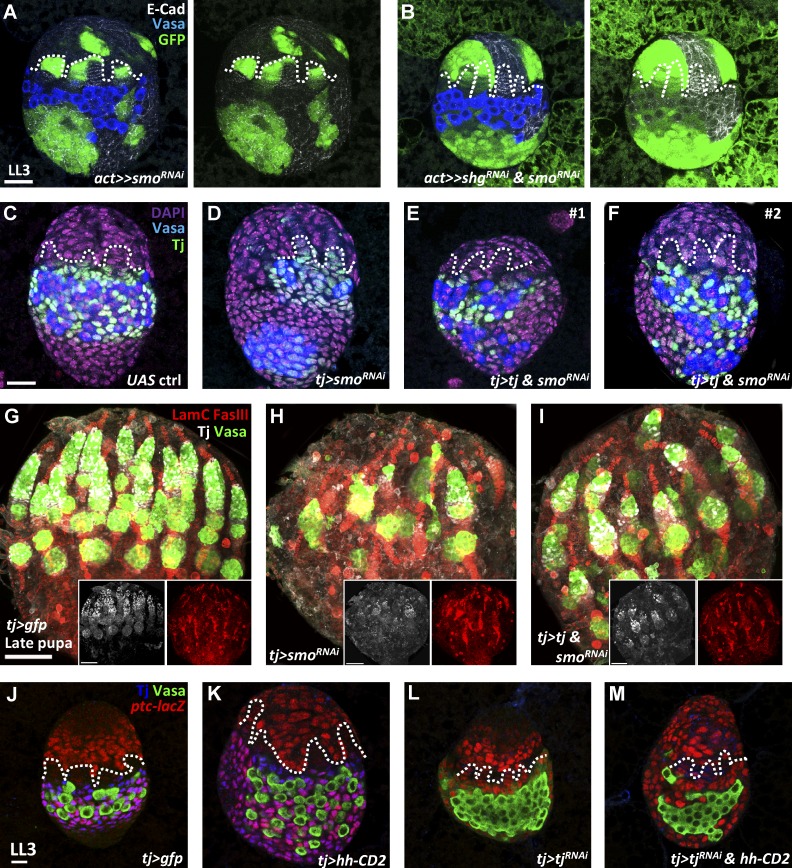
**Somatic Hh signaling controls IC formation to establish GSC niches and ovary morphogenesis.** (A and B) Late-L3 (LL3) *smo-*knockdown (A) and *smo- and shg-*knockdown (B) mosaic gonads with GFP (green, flip-out clones), E-cad (gray), and Vasa (blue, PGCs). (C–F) LL3 control (ctrl; C), *tj>smo^RNAi^* (D), and *tj>tj&smo^RNAi^* (E and F) gonads with Tj (green, ICs), Vasa (blue), and DAPI (magenta, DNA). Dashed lines mark forming TFs. (G–I) *tj>gfp* (G), *tj>smo^RNAi^* (H), and *tj>tj&smo^RNAi^* pupal ovaries (I) 72 h after puparium formation with LamC (red, TF and cap cell nuclear envelopes), FasIII (red, follicle and stalk cells), Tj (gray), and Vasa (green, germ cells). Insets in G–I with gray and red channels show that overexpression of *tj* in *smo*-knockdown ovaries partially restores GSC niche cells. (J–M) LL3 *tj>gfp* (J), *tj>hh-CD2* (K), *tj>tj^RNAi^* (L), and *tj> tj^RNAi^&hh-CD2* (M) gonads with *ptc-lacZ* (red, an Hh signaling reporter), Tj (blue), and Vasa (green). Knockdown *tj* expression in *hh*-overexpressing ovaries suppresses the ectopic formation of ICs and forms PGC clusters. Bars: (A and C) 20 µm; (G and insets) 50 µm; (J) 10 µm.

We next asked whether Hh signaling controls cell affinity for lineage specification via Tj–E-cadherin regulation. We first decreased E-cadherin expression in *smo*-knockdown somatic cells by using *flip-out* GAL4 to drive *shg^RNAi^* and *smo^RNAi^* and examined gonad morphogenesis at the late-L3 stage. *smo*-knockdown cells with increased E-cadherin expression adhered together (Fig. 8 A); strikingly, coknockdown *shg* and *smo* in somatic cells rescued the soma–germline interaction in 67% of gonads (*n* = 15; Fig. 8 B). In addition, compared with *tj>smo^RNAi^* gonads, overexpression of *tj* in *smo*-knockdown gonads by *tj-GAL4* partially restored soma–germline interaction accompanied by small PGC clusters (Fig. 8, C–F), suggesting that ICs are rescued. Consequently, overexpression of *tj* dramatically increased Tj-positive cells in *smo*-knockdown ovaries at the late pupal stage (Fig. 8, G–I). Furthermore, in addition to GSCs and niche cap cells, the ovariole number in 1-d-old *smo*-knockdown ovaries was partially rescued by supplying exogenous Tj (12.6 ± 3.8, *n* = 24) in comparison to control (16.6 ± 1.7, *n* = 22 ovaries, P < 0.001) and *tj>smo^RNAi^* ovaries (6.9 ± 2.4, *n* = 24 ovaries, P < 0.001; Fig. 8, G–I). Lastly, elimination of *tj* expression in *tj>hh* gonads suppressed ectopic formation of ICs and PGCs, which was observed in *tj>hh* gonads, and formed PGC clusters (Fig. 8, J–M). These results indicate that Hh signaling controls IC fate via E-cadherin mediated by Tj.

### Canonical Hh signaling directly activates *tj* transcription via Ci

**Figure 9.**
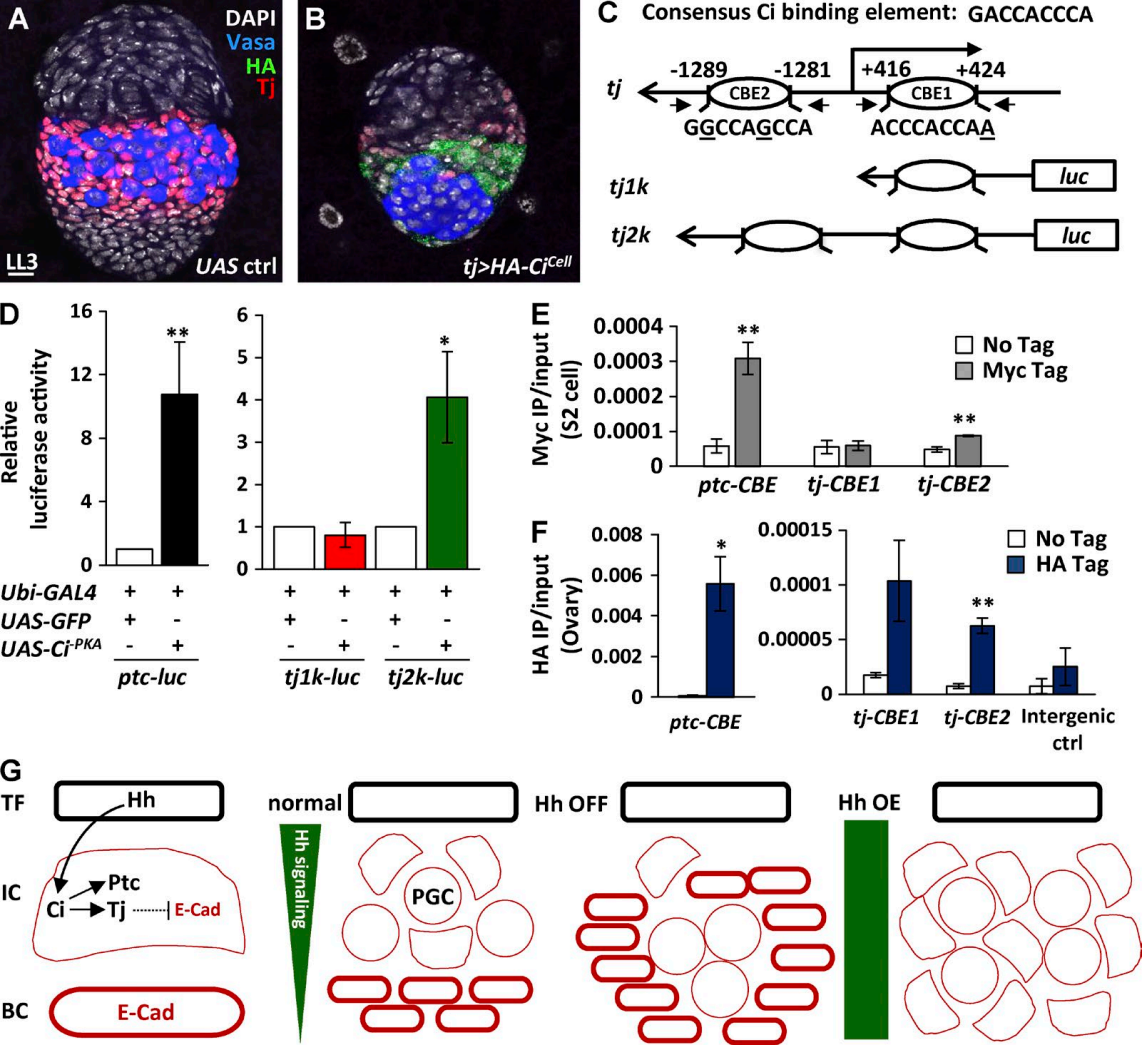
**Hh signaling activates *tj* transcription via Ci.** (A and B) Late-L3 control (ctrl; A) and *tj>HA-Ci^Cell^* gonads (B) with Tj (red, ICs), Vasa (blue, PGCs), DAPI (gray, DNA), and HA (green). Bar, 10 µm. (C) Luciferase expression driven by the 1 kb (*tj1k*) and 2 kb *tj* promoters (*tj2k*) carrying Ci-binding element 1 (CBE1), and both CBE1 and CBE2, respectively. (D) Luciferase reporter assay. S2 cells were transiently transfected with *ptc*, *tj1k*, or *tj2k* luciferase reporter plus *ubi-GAL4* together with *UAS-GFP* or *UAS-Ci^-PKA^*. The luciferase activity of *ubi-GAL4* & *UAS-GFP* was set at 1. (E and F) ChIP analysis of Ci binding in S2 cells (E) and in 1-d-old ovaries (F). The chromatin from S2 cells with or without expressing Myc-Ci^-PKA^ and ovaries with or without expressing HA-Ci^-PKA^ was precipitated with antibodies against Myc and HA, respectively. Coprecipitated DNA was analyzed by qPCR using primers against positions containing CBE of the *ptc* gene, CBE1, and CBE2 of the *tj* gene. The amplicon for the intergenic region was used as a negative control. Statistical differences in D–F were analyzed by two-tailed *t*-test. Error bars represent SD in D and SEM in E and F. *, P < 0.05; **, P < 0.001. (G) Hh signaling controls Tj via Ci to suppress E-cadherin (E-Cad) for IC fate. BC, basal cell; OE, overexpression.

Hh signaling regulates transcription through Ci, a sequence-specific DNA-binding protein (Von Ohlen et al., 1997), suggesting that Hh signaling controls *tj* transcription via Ci. To test this, we overexpressed a repressor form of Ci tagged with hemagglutinin, HA-Ci^Cell^ (Méthot and Basler, 1999), by *tj-GAL4* (Fig. 9, A and B). Similar to *tj>smo^RNAi^* gonads, *tj>HA-Ci^Cell^* gonads exhibited a large PGC cluster and decreased ICs (Tj positive) at the late-L3 stage. We also found two putative Ci-binding elements (CBEs) of the *tj* gene; CBE1 (AACCACCCA) was +424 to +416 bp in the 5′ untranslated region, and CBE2 (GGCCAGCCA) was −1,289 to −1,281 bp upstream of the transcriptional start site (Fig. 9 C). These CBEs were similar to the CBE (GACCACCCA) of the *ptc* promoter, which shows the highest Ci-binding affinity (Ramos and Barolo, 2013). We first examined the effects of Ci binding on *tj* transcription by using a promoter activity assay (Fig. 9 D). We generated luciferase reporter genes driven by 1.044 and 2 kb of the *tj* promoter containing CBE1 (*tj1k*) and CBE1 and CBE2 (*tj2k*), respectively. These reporters were transfected into S2 cells with *gfp* or *Ci^-PKA^*, a constitutive active form of Ci (Han et al., 2015), and were activated by *ubiquitin* (*ubi*)*-GAL4* (Fig. 9 D). The *ptc* promoter (−758 to +130 bp) containing a CBE was used as a positive control and exhibited an 11-fold increase of luciferase expression in cells with *ubi>Ci^-PKA^* compared with cells with *ubi>gfp*. The addition of Ci^-PKA^ increased luciferase expression by fourfold in cells transfected with *tj2k* reporter, but not *tj1k* reporter. We further used chromatin immunoprecipitation (ChIP) to examine whether Ci^-PKA^ (tagged with Myc) binds to the CBEs of the *tj* promoter in transfected or untransfected S2 cells (Fig. 9 E). We used qPCR to determine Ci^-PKA^ occupancy at the CBE of the *ptc* promoter (as a positive control). Antibodies against Myc efficiently immunoprecipitated the CBE of the *ptc* promoter at an amount sevenfold higher in S2 cells expressing Myc-tagged Ci^-PKA^ than in cells without transfection (negative control). The occupancy of Ci^-PKA^ in CBE2, but not CBE1, of the *tj* promoter showed a twofold enrichment compared with the negative control. ChIP assays with 1-d-old ovaries carrying *tj>HA-Ci^-PKA^* showed similar results (Fig. 9 E). This result was in agreement with a previous study showing that the low affinity of the Ci-binding site is critical for the transcription of several Hh target genes (Ramos and Barolo, 2013). Our results indicate that Ci activates *tj* transcription directly, predominately via interaction with CBE2.

## Discussion

The establishment of niches for stem cell recruitment, maintenance, and differentiation is a critical process during organ formation, but the mechanisms involved are unclear. We report that the fly ovary uses cell–cell adhesion via Hh signaling to select niche precursors (ICs), which express low levels of E-cadherin mediated by Tj and intermingle with PGCs (Fig. 9 G). Hh ligands are produced by TFs and are received by ICs, where Ptc is expressed and activated by Hh signaling, but not by basal cells, forming an Hh signaling gradient. Hh signaling activates Tj expression via Ci to suppress E-cadherin expression; in contrast, basal cells do not express Tj but have a high level of E-cadherin expression. When Hh signaling is disrupted, ICs become basal cells with reduced Tj and increased E-cadherin expression and do not intermingle with PGCs, leading to PGC clustering. Conversely, hyperactivation of Hh signaling activates Tj to suppress E-cadherin expression and converts all somatic cells posterior to TFs to ICs, which promotes PGC proliferation via unknown mechanisms. These findings add to our understanding of how organs use morphogen gradients to segregate cells for niche formation.

### Hh signaling in ovary development

Although Hh signaling participates in many aspects of organ development, few studies report the role of Hh signaling in ovary development. In *Drosophila*, Hh is expressed in the gonadal mesoderm, acting as a diffusible chemoattractant to guide PGC migration to the somatic gonad during embryogenesis (Deshpande et al., 2001; Araújo, 2015); however, some research results contradict this hypothesis (Renault et al., 2009). Sato et al. have proposed that TFs and PGCs in L3 ovaries produce Hh, which is received by PGCs to regulate PGC proliferation (Sato et al., 2010). In addition, Fused, a Hh signal–transducing serine/threonine kinase, is expressed in PGCs to mediate Hh signals produced by TFs for the control of soma–germline interaction (Besse et al., 2005). However, mutations of *smo* in germ cells do not show a similar phenotype (Besse et al., 2005). Here, we report the role of Hh signaling in determining ICs, which contribute to adult niche cap and escort cells. Hh signaling may also coordinate with Notch signaling to specify cap cells derived from the anterior of ICs adjacent to TFs. First, overexpression of Delta or Notch during development induces the transformation of escort cells to cap cells (Song et al., 2007), indicating that Delta produced by apical cells and TFs drives ICs to become cap cells. Second, Hh signaling occurs in forming cap cells (Fig. 1 O, inset), whereas Hh signaling revealed by *ptc-lacZ* is absent from adult cap cells (Liu et al., 2015b), suggesting that Hh signaling needs to be turned off in cap cells after they are specified. Third, overexpression of Hh signaling in gonadal somatic cells expands the IC population and induces ectopic escort cells, but not cap cells, at the adult stage; instead, escort cells occupy the space where cap cells are located, suggesting that constitutive activation of Hh signaling disrupts the specification of cap cells.

In the mouse ovary, folliculogenesis occurs when an individual oocyte is surrounded by epithelial granulosa cells that recruit precursors of stromal theca cells for steroid production (Hirshfield, 1991). A recent paper also reported that specification of theca cells requires expression of Gli1 (Ci orthologue in mammals) in response to Hh from granulosa cells (Liu et al., 2015a). In addition, Tj is most similar to mammalian c-Maf and Maf B based on their high similarity in amino acid sequence and protein structure (Li et al., 2003). Large Maf proteins act as major regulators for cell differentiation in many tissues (Tsuchiya et al., 2015). Although the function of Maf proteins in mammal ovary is unexplored, c-Maf and MafB are expressed in stromal cells of mouse embryonic ovaries (Maatouk et al., 2012). These results suggest a conserved role of Hh signaling to large Maf transcription factors in the lineage determination of ovarian somatic cells. Furthermore, ovarian cell hyperproliferation induced by Hh (Fig. 4) or Tj overexpression (not depicted) is reminiscent of the mechanism by which aberrant activation of Hh signaling causes ovarian cancer (Russell et al., 2007).

### Differential cell affinity in organ development

During tissue or organ development, the cell fate decision is followed by segregation of cells into distinct domains, where different cell lineages are further specified (Krens and Heisenberg, 2011). A general hypothesis is that tissue separation occurs through differential adhesion of cells (Fagotto, 2014). The most accepted experimental results to support this hypothesis involve the reformation of well-segregated cell populations from a mixed aggregate initially formed by cells that are dissociated from different embryonic tissues (Moscona and Moscona, 1952; Steinberg and Gilbert, 2004). These results also suggest that cells have the ability to recognize the identity of their neighbors and gather with cells of the same type. Several classic examples in both vertebrate and invertebrate systems have been used as paradigms for the analysis of tissue separation (Fagotto, 2014), such as embryonic parasegment boundaries and anterior–posterior and dorsoventral compartments of larval wing discs in *Drosophila*, as well as the formation of germ layers, somites, and rhombomeres in frog and fish. However, many other segregations in developing tissues have not been investigated, such as that of the ovary. Here, we report that the development of the *Drosophila* ovary requires such separation to specify stromal ICs and epithelial basal cells and that this process is controlled by an Hh signaling–Tj–E-cadherin axis. Interestingly, in the *Drosophila* wing disc, the regulation of cadherin Cad 99 by of Hh signaling is involved in setting the anterior–posterior compartment (Schlichting et al., 2005). In addition, Sonic Hh signaling negatively regulates E-cadherin expression in mouse gastric epithelial cells in the culture system (Xiao et al., 2010). These results suggest a common role for Hh signaling in the control of cell adhesion and its general participation in tissue separation.

## Materials and methods

### *Drosophila* strains and culture

*Drosophila* stocks were maintained at 22–25°C on standard medium, unless otherwise indicated. *w^1118^* was used as a wild-type control. The following fly strains were used in this study: cold-sensitive mutant *smo^3^* and hypomorphic allele *ptc^S2^* have been described previously (Chen and Struhl, 1996). *ptc-lacZ* was used to monitor Hh signaling (Chen and Struhl, 1996). *PZ1444* (a gift from T. Xie, Stowers Institute for Medical Research, Kansas City, MO) and *MA33* (a gift from T. Schüpbac, Princeton University, Princeton, NJ) are enhancer trap lines in which lacZ is expressed in niche cap and escort cells (Margolis and Spradling, 1995; Kirilly et al., 2011) and in ICs (Gilboa and Lehmann, 2006), respectively. *UAS-RNAi* lines against *smo* (BL43134 and NIG-Fly 11561R-1), *ptc* (NIG-Fly 2411R-1), *tj* (Vienna *Drosophila* RNAi Center [VDRC] 30526, second chromosome [II], and 108255, third chromosome [III]), *shg* (VDRC 27081), and Ci (BL31320, BL28984, NIG-Fly 2125R-1, and VDRC 105620) were obtained from the VDRC, the National Institute of Genetics-Fly Stocks, or the Bloomington *Drosophila* Stock Center (BL). Sequences of the RNAi lines used in this study are available from the website of each stock center, and their efficiencies were described previously or tested here. In this study, we used the *smo^RNAi^* line from Bloomington, unless otherwise indicated. *UAS-hh-gfp*, *UAS-hh-CD2*, *UAS-tj* (a gift from D. Godt, University of Toronto, Toronto, Canada), *UAS-HA-piwi* (a gift from R. Lehmann, New York University School of Medicine, New York, NY), *UAS-HA-Ci^Cell^* (a gift from C.-T. Chien, Institute of Molecular and Cell Biology, Academia Sinica, Taipei, Taiwan), *UAS-HA-Ci^-PKA^* (a gift from J. Jiang, UT Southwestern Medical Center, Dallas, TX)*, tj-GAL4*, *bab1-GAL4, ptc-GAL4*, and *hh-GAL4* lines (a gift from M. Buszczak, UT Southwestern Medical Center, Dallas, TX) were described previously (Burke et al., 1999; Méthot and Basler, 1999; Tanimoto et al., 2000; Torroja et al., 2004; Bolívar et al., 2006; Hsu and Drummond-Barbosa, 2009; Gunawan et al., 2013; Tseng et al., 2014). Other genetic elements are described in FlyBase (http://flybase.bio.indiana.edu).

### Developmental stage of larvae and pupae

Morphological definitions of the developmental stages of *Drosophila* followed those of Ashburner (2005). Flies were transferred into a fresh vial to lay eggs at 25°C for 3 h and were then removed. The vials were left at 25°C. After hatching (∼20 h after egg laying [AEL]), larvae were collected as first-instar larvae (L1). At this stage, SGPs intermingled with PGCs and did not form specific groups. We defined larvae with gonads exhibiting apical gonadal somatic cells as early second-instar larvae (early L2; ∼50 h AEL), larval gonads starting to form TFs as late-L2 (∼70 h AEL), and larvae that climbed up and down from the food as mid-third instar larvae (mid-L3; ∼100 h AEL). We defined the larvae that moved out of food but in which pupation had not started as late-L3 wandering larvae (∼125-140 h AEL). At this stage, most TFs were still forming and cap cells were starting to form. To obtain pupae that were at a synchronized developmental stage, we removed wandering larvae but kept white prepupae (referred to as 0 h prepupae) in the vial.

In addition to synchronization at hatched L1 and white prepupal stages, we also used gonadal somatic cell development as an indicator to stage the larval and pupal gonads of flies at 18°C and 29°C (Fig. 1).

### Gal4-based cell lineage analysis

G-TRACE was used to trace lineages of GAL4-expressing cells (Evans et al., 2009). The embryos that were produced by crosses carrying the G-TRACE cassette (*UAS-DsRed, UAS-flp; ubi>Stop>gfp/CyO*) (a gift from H. Sun, Institute of Molecular and Cell Biology, Academia Sinica, Taipei, Taiwan) and flies carrying *tub-GAL80^ts^* with *hh-GAL4*, *ptc-GAL4,* or *tj-GAL4* were collected within 3 h at 25°C. The embryos were cultured at 18°C until dissection (1 day after eclosion), except for the stage-specific heat shock at 29°C to induce FLP. For embryo heat shock, collected embryos were cultured at 29°C until hatching (∼16 h). To perform larval-stage shock, we cultured embryos at 18°C until hatching (∼37 h AEL) and collected newly hatched larvae (L1) within 30 min for stage synchronization. For L1 and L2 shocks, newly hatched L1 larvae (∼85 h AEL) were switched to 29°C for 24 h. For L3 shock, mild-L3 larvae that remained in the food but were climbing up and down from the food (∼135 h AEL) were switched to 29°C for 17 h. For pupa stage shock, white pupae (∼217 h AEL) were switched to 29°C until eclosion.

### Genetic mosaic analysis

Genetic mosaics were generated by FLP/FRT-mediated mitotic recombination (Xu and Rubin, 1993). For conventional mosaic analysis, L1 larvae of the genotypes *hs-flp^122^/+; smo^3^ FRT40A/ubi-gfp FRT40A*, *hs-flp^122^/+; ubi-gfp FRT40A/neoFRT40A*, *hs-flp^122^/+*; *FRT42D ptc^S2^/FRT42D ubi-gfp*, and *hs-flp^122^/+; FRT42D arm-lacZ/FRT 42D ubi-gfp* were generated from standard crosses and subjected to heat shock for 1 h at 37°C, twice a day for 3 d. After heat shock, the larvae were cultured at 25°C until late L3 for dissection (*smo^3^* mosaic mutant larvae were cultured at 18°C). For flip-out clone analysis, L1 larvae of genotypes *hs-flp^122^/+; ptc-lacZ/+; UAS-smo^RNAi^/actin5c>Stop>GAL4 UAS-gfp, hs-flp^122^/+; UAS-ptc^RNAi^/actin5c>Stop>GAL4 UAS-gfp, hs-flp^122^/+; UAS-tj^RNAi^/actin5c>Stop>GAL4 UAS-gfp, hs-flp^122^/+; UAS-tj/+; actin5c>Stop>GAL4 UAS-gfp/+, hs-flp^122^/+; UAS-shg^RNAi^/+; UAS-smo^RNAi^/actin5c>Stop>GAL4 UAS-gfp* and *hs-flp^122^*/+; *actin5c>Stop>GAL4 UAS-gfp/+* were generated from standard crosses and subjected to heat shock for 10 min at 37°C. After heat shock, the larvae were cultured at 25°C until dissection. Homozygous mutant cells were identified by the absence of GFP in conventional mosaic analyses, and RNAi-expressing cells were recognized by the presence of GFP in flip-out clones.

### Quantitative real-time PCR

Total RNA of male or female gonads of late-L3 larvae (∼25) was extracted with TRIzol reagent (Invitrogen). 1 µg total RNA was reverse transcribed with the Transcriptor First Strand cDNA Synthesis kit (Roche). Steady-state mRNA levels were determined by using the LightCycler 480 Probes Master combined with a Universal Probe library (Roche).

Each gene was analyzed using the primer pairs and probes listed below: *tj*: probe#52, 5′-CTGAACAAGCGGCTCCAT-3′ and 5′-CGTCGCTTCTGCTTCAGAC-3′; *piwi*: probe#54, 5′-CTGCCCGAGAGATACGACTT-3′ and 5′-CGACACTGTACCCTGACGAA-3′; *fasIII*: probe #101, 5′-TTGACACAAAACACATCCTCTACA-3′ and 5′-TTGATTTAATGTGTGGGCTGA-3′; *ptc*: probe#73, 5′-AGCCTAAGCCGTAACCCTATTT-3′ and 5′-CCATGAGAATCCATGAGAACG-3′; *phf7*: probe#15, 5′-CTGTTGGGCTCCATCTCG-3′ and 5′-AGTGCGGCGACGTAAGAC-3′; *RpL19*: probe #128, 5′-GAGCGTATTGCCACCAGGA-3′ and 5′-CGATCTCGTCCTCCTTAGCA-3′; *RpL32*: probe #117, 5′-CGGATCGATATGCTAAGCTGT-3′ and 5′-CGACGCACTCTGTTGTCG-3′.

### Generation of *Drosophila tj* promoter constructs

We amplified 1.044 kb (*tj1K*, position −555 to +489) and 2 kb (*tj2K*, position −1,511 to +489) of the *Drosophila tj* promoter before the ATG translation start site containing one and two putative CBEs (−1,289 to −1,281 and +416 to +424), respectively, from a BAC clone containing the *tj* gene (RP98-28G24, BACPAC Resources Center) by PCR using the following primers: *tj2K* 5′-GGGGGG(KpnI site)ATACGAGCCAAAACAAATCG-3′ and 5′-GGGGGG(XhoI site)TGGATCGACCAGGGAC-3′; *tj1K* 5′-GGGGGG(KpnI site)GCAGATCAAAATATAATCGC-3′ and 5′-GGGGGG(XhoI site)TGGATCGACCAGGGAC-3′. The KpnI and XhoI sites were used to insert *tj* promoter fragments upstream of the firefly luciferase reporter gene in pGL4.15 (Promega).

### Luciferase reporter assay

*Drosophila* S2 cells were cultured with Schneider’s *Drosophila* medium, containing 10% FBS and 10% streptomycin/penicillin. *tj1K* and *tj2K* of the *tj* promoter region were cloned into the pGL 4.15 vector. *ptc-luciferase* and *RL-PolIII Renilla* luciferase reporters were used as positive and internal controls, respectively. A total of 2.7 × 10^6^ S2 cells were transfected with 1.1 µg reporter constructs (1 µg *Luciferase* and 0.1 µg *Renilla*) and 1 µg expression constructs (0.5 µg *ubi-GAL4* and 0.5 µg *UAS-GFP* or *UAS-Myc-Ci^-PKA^*; a gift from J. Jiang) using Cellfectin II reagent (Invitrogen). After being transfected for 5 h, the cells were split into a 24-well plate and cultured for 72 h at 25°C. Luciferase activity was subsequently measured with a Dual-Glo luciferase assay kit (Promega). Firefly luciferase activity was normalized to *Renilla* luciferase activity. Data represent the mean ± SD of at least three independent experiments.

### ChIP

ChIP assays were performed as previously described (Huang et al., 2014), with minor modification. S2 cells (2.5 × 10^6^) were seeded per well in six-well plates and transfected with 1 µg expression constructs (0.5 µg *ubi-GAL4* and 0.5 µg *UAS-Myc-Ci^-PKA^*) 1 day after seeding using Effectene transfection reagent (QIAGEN). Cells that were not transfected were used as the background control. At 48 h after transfection, the cells were harvested and fixed in Schneider’s insect medium containing 10% FBS and 1% formaldehyde for 10 min at room temperature; cross-linking was stopped by adding glycine to a final concentration of 125 mM. After two washes with PBS, the fixed cells were sonicated in 300 µl buffer A2 (15 mM Hepes, pH 7.5, 140 mM sodium chloride, 1 mM EDTA, 0.5 mM EGTA, 1% TritonX-100, 0.1% sodium deoxycholate, 0.1% SDS, 0.5% N-lauroyl sarcosine, 1 mM PMSF, 5 mM sodium fluoride, 5 mM sodium butyrate, and protease inhibitor cocktail; Roche) using a Bioruptor (Diagenode) for 15 min (30 s on/30 s off) on the high-power setting. After centrifugation at 4°C, 7.5 µl soluble chromatin was subjected to Western blot to check transfection, and 240 µl soluble chromatin was incubated with 1 µl mouse antimyc (9B11; Cell Signaling Technology) and 5 µl mouse antimyc (9E10; Santa Cruz Biotechnology, Inc.) antibodies overnight at 4°C. Antibody-bound chromatin was pulled down by magnetic protein A/G beads (EMD Millipore) for 2 h at 4°C, and the beads were washed five times with RIPA buffer (50 mM Hepes, pH 7.5, 1 mM EDTA, 0.7% sodium deoxycholate, 1% NP-40, 0.5 M LiCl, and 1 mM PMSF) and once with TE buffer containing 50 mM NaCl. The chromatin was eluted twice in TE buffer containing 1% SDS and 250 mM NaCl for 20 min at 65°C. After RNase A and proteinase K treatment, cross-linking was reversed overnight at 65°C. DNA was purified by phenol-chloroform extraction and ethanol precipitation. The quantitative PCR data obtained from the input and immunoprecipitated DNA samples were analyzed with the standard curve method.

ChIP of ovaries was performed with 180 pairs of ovaries from 1-d-old flies carrying *UAS-HA-Ci^-PKA^* (no tag control) alone or *tj-GAL4 and UAS-HA-Ci^-PKA^*. Ovaries were dissected in Shield insect medium and fixed in 950 µl PBS containing 1.8% formaldehyde for 10 min at room temperature. Crossing-linking was stopped by adding 50 µl 2.5 M glycine and incubating for 5 min. After two washes with PBS, fixed cells were sonicated in 400 µl buffer A2 at the same sonicator setting. After centrifugation, 280 µl soluble chromatin was incubated with 5 µl rat anti-HA (3F10; Roche) overnight at 4°C and processed for ChIP analysis.

The following primers were used to amplify fragments of the *tj* or *ptc* gene: *tj-CBE1* (327–569 bp): 5′-TCTGTACTCCGTTTCCGCTG-3′ and 5′-AATTCCCGCATAGCCCCATT-3′; *tj-CBE2* (−1,459 to −1,240 bp): 5′-AAGCGCCAAAGTGCGGATA-3′ and 5′-CCATTTCCGCCCACCTCC-3′; *ptc CBE* (−778 to −693 bp): 5′-AGCTGAACGTTTGGGTAGGG-3′ and 5′-CAAATAGCTCCGCCACGAGA-3′; intergenic control: 5′-GAGCAGACAACGCTCCAAGACCCAA-3′ and 5′-AAATTTTCCACCTACCTGCCGCACG-3′. The amplicon for the intergenic region is located at chr2L (18,415,855–18,415,978). Error bars represent SEM (*, P < 0.05; **, P < 0.001).

### Immunostaining and fluorescence microscopy

Adult ovaries were dissected, fixed, and immunostained as described previously (Hsu et al., 2008). In brief, ovaries were dissected in Grace’s insect medium (Lonza) and fixed with 5.3% paraformaldehyde/Grace’s insect medium for 13 min with gentle agitation at room temperature. Ovaries were washed in PBST (0.1% Triton X-100 in PBS) 20 min for three times, and teased apart in PBST then incubate with blocking solution (5% bovine serum albumin and 0.05% normal goat serum in PBST) for 3 h at room temperature or 4°C overnight. Ovaries were incubated with primary antibodies (diluted in blocking solution) for 3 h at room temperature or 4°C overnight, and follow by PBST 30 min wash for three to four times. Then ovaries were incubated with secondary antibodies (diluted in blocking solution) for 3 h at room temperature or 4°C overnight, and follow by PBST 30 min wash for three to four times. For gonad dissection, larvae were transferred into a clear glass well, and their gonads and associated fat body were dissected in Grace’s insect medium (Lonza). The gonads and associated fat body were transferred into a 24-well cell culture dish with mesh inserts and fixed with 5.3% paraformaldehyde at room temperature for 13 min with gentle agitation. After washing, gonads were immunostained following a standard procedure, as described above. The following primary antibodies were used: rabbit anti-Hh (1:1,000; a gift from T. Tabata, Institute of Molecular and Cellular Biosciences, Tokyo, Japan), guinea pig anti-Tj (1:5,000; a gift from D. Godt), mouse anti-Piwi (1:500; a gift from M. Siomi, University of Tokyo, Tokyo, Japan), mouse anti–Hu-li tai shao (*Drosophila* adducing-related protein; 1:10; 1B1, Developmental Studies Hybridoma Bank [DSHB]), rat anti-Ci (1:10; 2A1; DSHB), mouse anti–Lamin C (1:25; LC28.26; DSHB), mouse anti-Ptc (1:100; DSHB), mouse anti-FasIII (1:50; 7G10; DSHB), rat anti–DE-cadherin (1:3; DCAD2; DSHB), rabbit anti-Vasa (1:500; Santa Cruz Biotechnology, Inc.), mouse anti–β-gal (1:1,000; Promega), rabbit anti-GFP (1:2,000; Torrey Pines), mouse anti-Myc (1:50; 9E10; Santa Cruz Biotechnology, Inc.), rat anti-HA (1:200; 3F10; Roche), rabbit anti–phospho (p)-ErK (1:200; 4370; Cell Signaling Technology), and rabbit anti-pMad (phospho S423+S425; 1:100; 52903; Abcam). Note that although Ci transcription was detected in every cell of the late-L3 gonad examined by *ci-lacZ* (Sato et al., 2010), we did not detect Ci expression in the larval gonad by using anti–Ci antibody (unpublished data), whose specificity has been demonstrated (Chen et al., 2000; Oh et al., 2015), probably because of its low expression. Alexa Fluor 488–, Alexa Fluor 568–, or Alexa Fluor 633–conjugated goat anti–mouse, anti–rabbit, anti–rat, and anti–guinea pig secondary antibodies (1:400; Molecular Probes or Abcam) were used. F-actin was stained by phalloidin (1:80, R415; Thermo Fisher Scientific). Samples were stained with 0.5 µg/ml DAPI (Sigma-Aldrich), mounted in 80% glycerol containing 20.0 µg/ml *N*-propyl gallate (Sigma-Aldrich), and analyzed using LSM 700 confocal microscopes (ZEISS).

### Microscopy

Ovary or gonad samples were stored in mounting solution (80% glycerol containing 20.0 µg/ml *N*-propyl gallate) at 4°C before mounting. The immunostaining signals were detected using an upright confocal system (LSM700) with Axio imager 2 microscope (ZEISS) at 21–22°C. Confocal images were captured by the upright confocal system with a Plan-APOCHROMAT 63×/1.4 NA or 40×/1.3 NA oil objective lens and the acquisition software ZEN Image Brower (ZEISS). The images were explored and then processed in Photoshop 7.0.1 and Illustrator CS6 (Adobe Systems).

### Statistical and quantification analyses

For GSC and cap cell analyses, GSCs were identified by the anterior position of their fusome (recognized by 1B1 labeling), which is juxtaposed to cap cells whose nuclear envelopes are ovoid and recognized by LamC labeling. For escort cell counting, we counted germarial somatic cells that were positive for *ptc-lacZ* or *PZ1444* lines but negative for LamC (cap cells) and FasIII (follicle cell lineage). All statistical data were recorded in Excel (Microsoft) and graphed in either Excel or Prism 6.0 (GraphPad Software). p-values were calculated using two-sided unpaired *t* tests or χ^2^ in Excel or Prism. P < 0.05 was considered a statistically significant difference. Error bars represent the SD or SEM as described in the figure legends. For analyzing pMad expression in PGCs and Tj and Piwi expression in GFP-negative ICs of *smo^3^* mutant mosaic gonads, ImageJ was used to measure the mean fluorescence intensity (arbitrary units) in confocal z-sections at the largest IC cell nuclear diameter. Tj and Piwi expression in GFP-negative ICs neighboring GFP-positive ICs were also measured as references. For IC and PGC counting, z-sections covering a whole gonad were reconstructed into a 3D image, and the numbers of Tj-positive cells or PGCs were analyzed by Imaris ×64 8.4.0 (Bitplane). For experiments analyzed with *t* tests, data distribution was assumed to be normal, but this was not formally tested.

### Online supplemental material

Fig. S1 shows *G-trace* activated by *hh-GAL4*, *ptc-GAL4*, or *tj-GAL4* in ovaries at different developmental stages. Fig. S2 shows that knockdown somatic Hh signaling decreases ICs accompanied by PGC clusters. Fig. S3 shows that somatic Hh signaling controls niche formation, GSC recruitment, and maintenance. Fig. S4 shows that suppression of somatic Hh signaling does not induce cell death, decrease Egfr signaling, or decrease Piwi expression. Fig. S5 shows that overexpression of *tj* in *smo*-knockdown gonads partially rescues ovary morphogenesis, niche cap cells, and GSC numbers.

## Supplementary Material

Supplemental Materials (PDF)
