# Supplementary material for: Hedgehog signaling establishes precursors for germline stem cell niches by regulating cell adhesion
Source: J Cell Biol. 2017 May 1;216(5):1439–53. doi: 10.1083/jcb.201610063 (PMC5412570; doi:10.1083/jcb.201610063)
Supplement: Supplemental Materials (PDF) [file JCB_201610063_sm.pdf]

Lai et al., https://doi.org/10.1083/jcb.201610063

THE JOURNAL OF CELL BIOLOGY

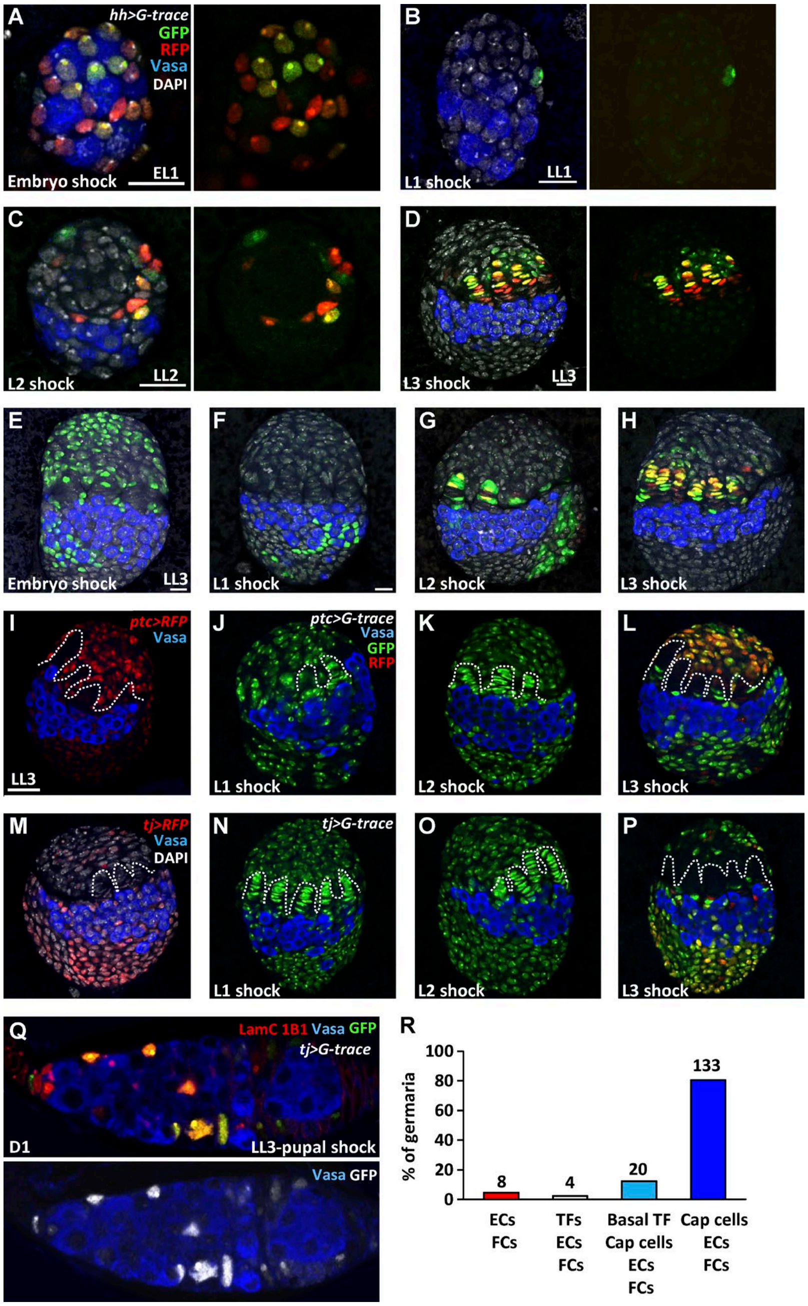

Figure S1. ***G-trace*****activated by *hh-GAL4*, *ptc-GAL4*, or *tj-GAL4* in ovaries at different developmental stages.** (A–D) early L1 (A), late-L1 (B), late-L2 (C), and late-L3 gonads (D) carrying *hh>G-trace* activated at the embryo, L1, L2, and L3 stages, respectively. Late-L1 gonads carried very few GFP-positive cells, probably because the low expression of *hh-GAL4* at the L1 stage. (E–H) Late-L3 gonads carrying *hh>G-trace* activated at the embryo (E), L1 (F), L2 (G), and L3 (H) stages. (I) *ptc-GAL4* expression in apical cells and ICs of the late-L3 gonad. (J–L) Late-L3 gonads carrying *ptc>G-trace* activated at the L1 (J), L2 (K), and L3 (L) stages. (M) *tj-GAL4* expression in ICs and basal cells of the late-L3 gonad. (N–P) Late-L3 gonads carrying *tj>G-trace* activated at the L1 (N), L2 (O), and L3 (P) stages. (Q) One-day-old (D1) germarium carrying *tj>G-trace* activated during the late-L3 to pupal stages. RFP represents real-time expression of each GAL4, GFP represents the GAL4-expressing cell lineage, and Vasa labels PGCs or germ cells. DAPI in A–H and M labels DNA. Dashed lines mark terminal filament. Bars, 10 µm. (R) Percentage of 1-d-old *tj>G-trace* germaria with GFP, induced during the late-L3 to pupal stage, in different groups of germarial somatic cells. ECs, escort cells; FCs, follicle cells. Numbers of germaria analyzed are shown above each bar.

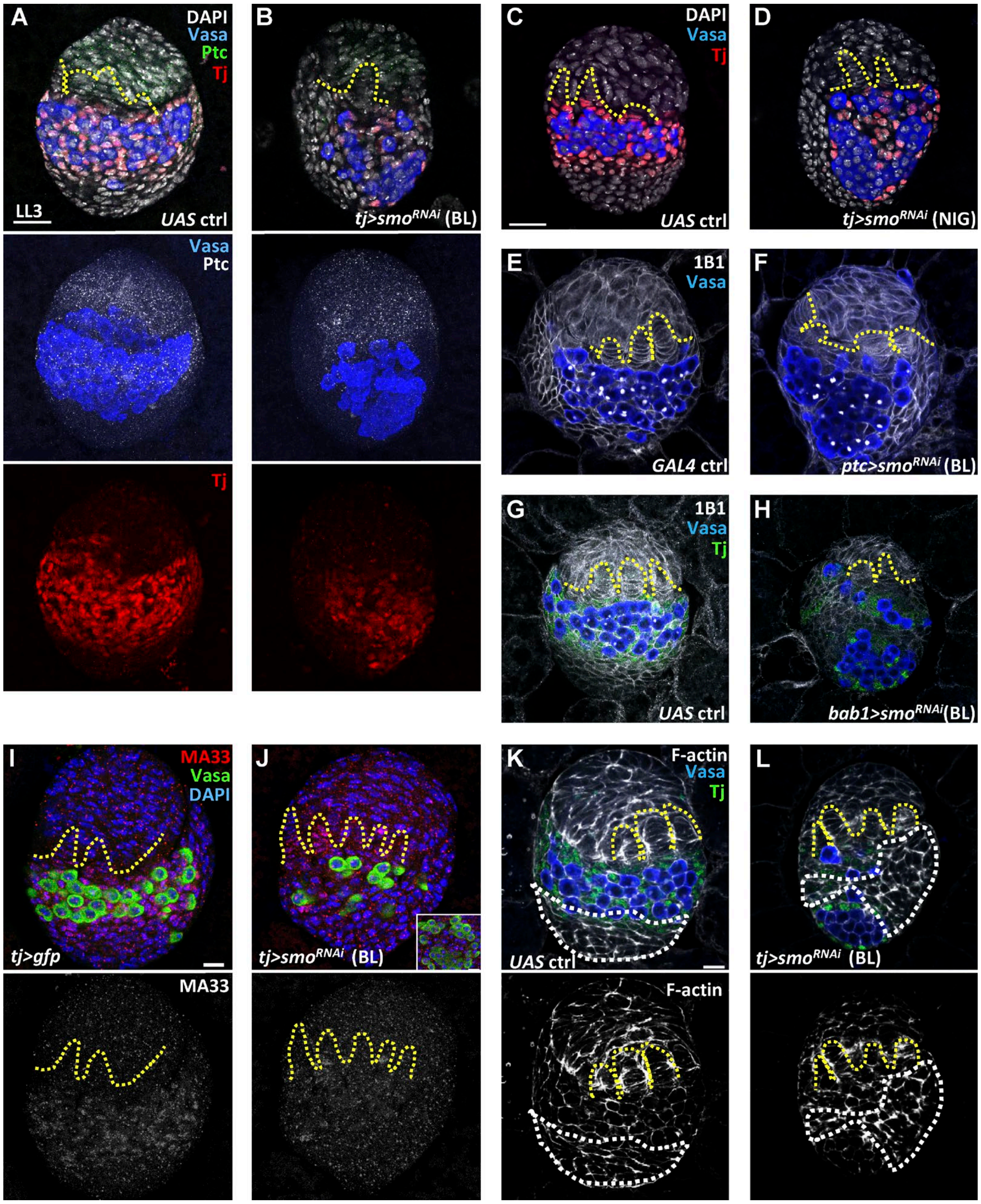

Figure S2. **Knockdown somatic Hh signaling decreases ICs accompanied by PGC clusters.** (A and B) Knockdown *smo* by *tj-GAL4* causes reduction of ICs and PGCs. Late-L3 *UAS* control (ctrl; A) and *tj>smo*^{RNAi} (BL; B) gonads with Ptc (green in A, top, and B, top; gray in A, middle, and B, middle), Tj (red, ICs), Vasa (blue, PGCs) and DAPI (gray, DNA). (C–H) Knockdown smo using *tj-GAL4* to drive a different *smo*^{RNAi} line (NIG), or using *ptc-GAL4* or *bab1-GAL4* to drive the same *smo*^{RNAi} line (BL) causes PGC clusters. (C and D) Late-L3 control (ctrl; C) and *tj>smo*^{RNAi} (NIG) gonads (D) with Tj (red), Vasa (blue), and DAPI (gray). (E and F) Late-L3 *ptc-GAL4* control (E) and *ptc>smo*^{RNAi} (BL; F) gonads with 1B1 (gray, germ cell fusomes and cell membranes) and Vasa (blue). (G and H) Late-L3 control (ctrl; G) and *bab1>smo*^{RNAi} (BL) gonads (H) with Tj (green), 1B1 (gray), and Vasa (blue). BL, Bloomington stock center; NIG, The National Institute of Genetics-Fly Stocks. (I–L) ICs of *tj>smo*^{RNAi} gonads display basal cell characteristics. Late-L3 *tj>gfp* (I and K) and *tj>smo*^{RNAi} (BL) gonads (J and L) with *MA33LacZ* (red in I and J, an enhancer trap expressed in ICs), F-actin (gray in K and L), Vasa (green in I and J, blue in K and L), and DAPI (blue in I and J). ICs of *tj>smo*^{RNAi} gonads express very low levels of MA33 but express F-actin comparable to that in basal cells. Inset in J shows PGC cluster in different focal plane. Yellow dashed lines mark terminal filament; white dashed circles outline basal cells in K and basal cell-like cells in L. Bars, 10 µm.

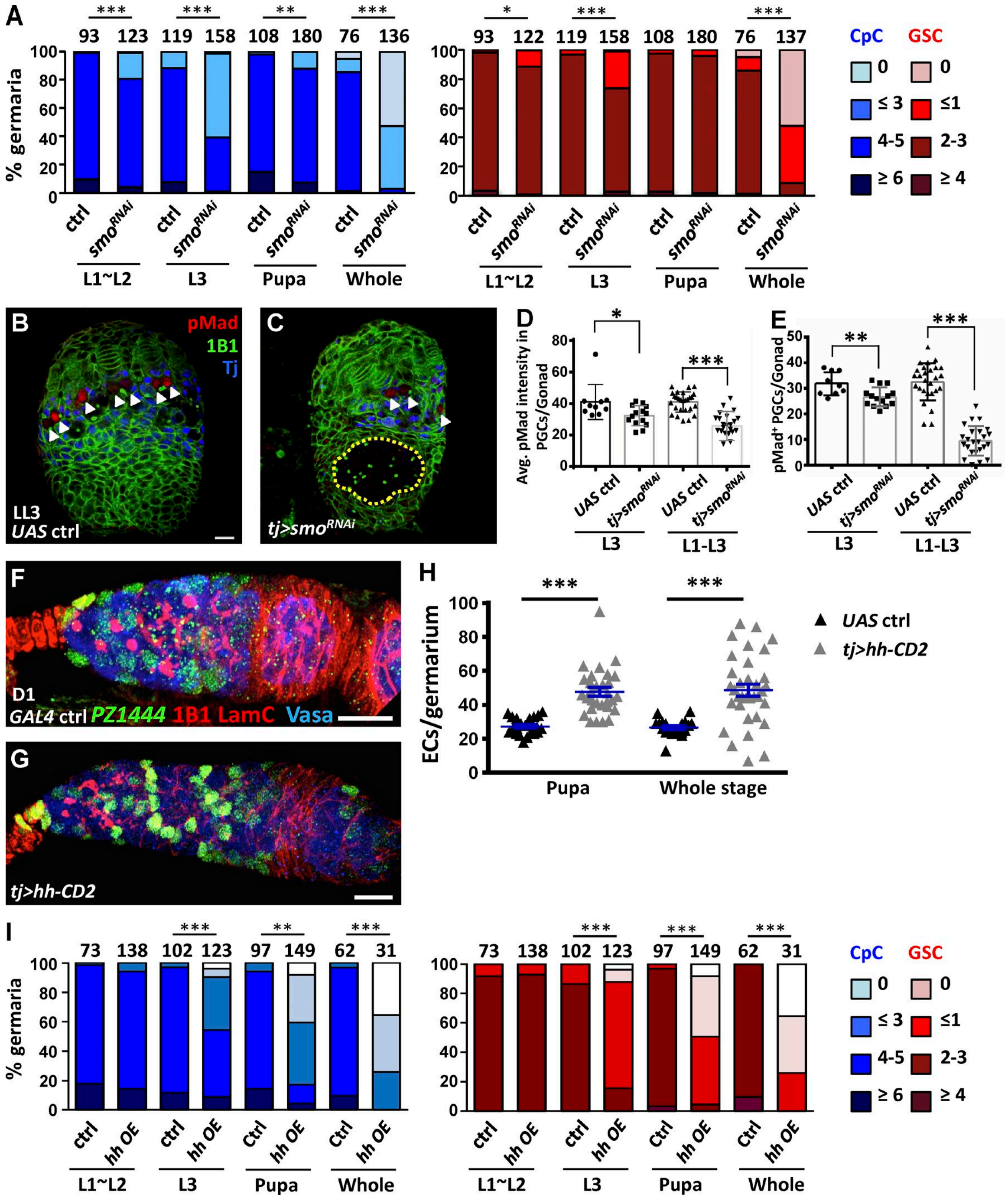

Figure S3. **Somatic Hh signaling controls niche formation, GSC recruitment, and maintenance.** (A) Disruption of somatic Hh signaling decreases niche cap cells, which causes GSC loss. (A) Cap cell (blue bars) and GSC (red bars) numbers in 1-d-old control (ctrl) and *tj>smo*^{RNAi} germaria, in which *tj-GAL4* was activated at different developmental stages. (B–E) Knockdown somatic smo reduces GSCs in late-L3 gonads. Late-L3 control (ctrl; B) and *tj>smo*^{RNAi} (C) gonads with phospho (p)-Mad (red, GSCs marked by arrowheads), Tj (blue, ICs), and 1B1 (green, PGC fusomes and somatic cell membrane). Dashed circle outlines PGC cluster. Bar, 10 µm. Mean (avg.) pMad intensity in GSCs (D) and GSC number (E) per gonad of late-L3 control and *tj>smo*^{RNAi} larvae, in which *tj-GAL4* was activated at indicated stages. (F–I) Overexpression of Hh in somatic gonadal precursors induces ectopic niche escort cells but decreases niche cap cells and GSCs. One-day (D)-old control (ctrl; F) and *tj>hh-CD2* (G) with *PZ1444* LacZ (green, escort cells), Vasa (blue, germ cells), 1B1 (red, fusome and follicle cell membranes), and LamC (red, TF and cap cell nuclear envelopes). Bars, 10 µm. (H) Escort cell (EC) number per germarium of newly eclosed control and flies with *hh-CD2* overexpression driven by *tj-GAL4* from pupal to adult or throughout development (whole stage). (I) Cap cell (blue bars) and GSC (red bars) numbers in germaria of newly eclosed control and flies with *hh-CD2* overexpression (*hh OE*) driven by *tj-GAL4* at indicated developmental stages. L1, L2, and L3 are first-, second-, and third-instar larval stages, respectively; whole, whole developmental stages. Numbers of germaria analyzed are shown above each bar. Statistical differences were analyzed by χ^2 in A and I and by two-tailed *t* test in in D and H. Error bars represent SEM. *, P < 0.05; **, P < 0.01; ***, P < 0.001.

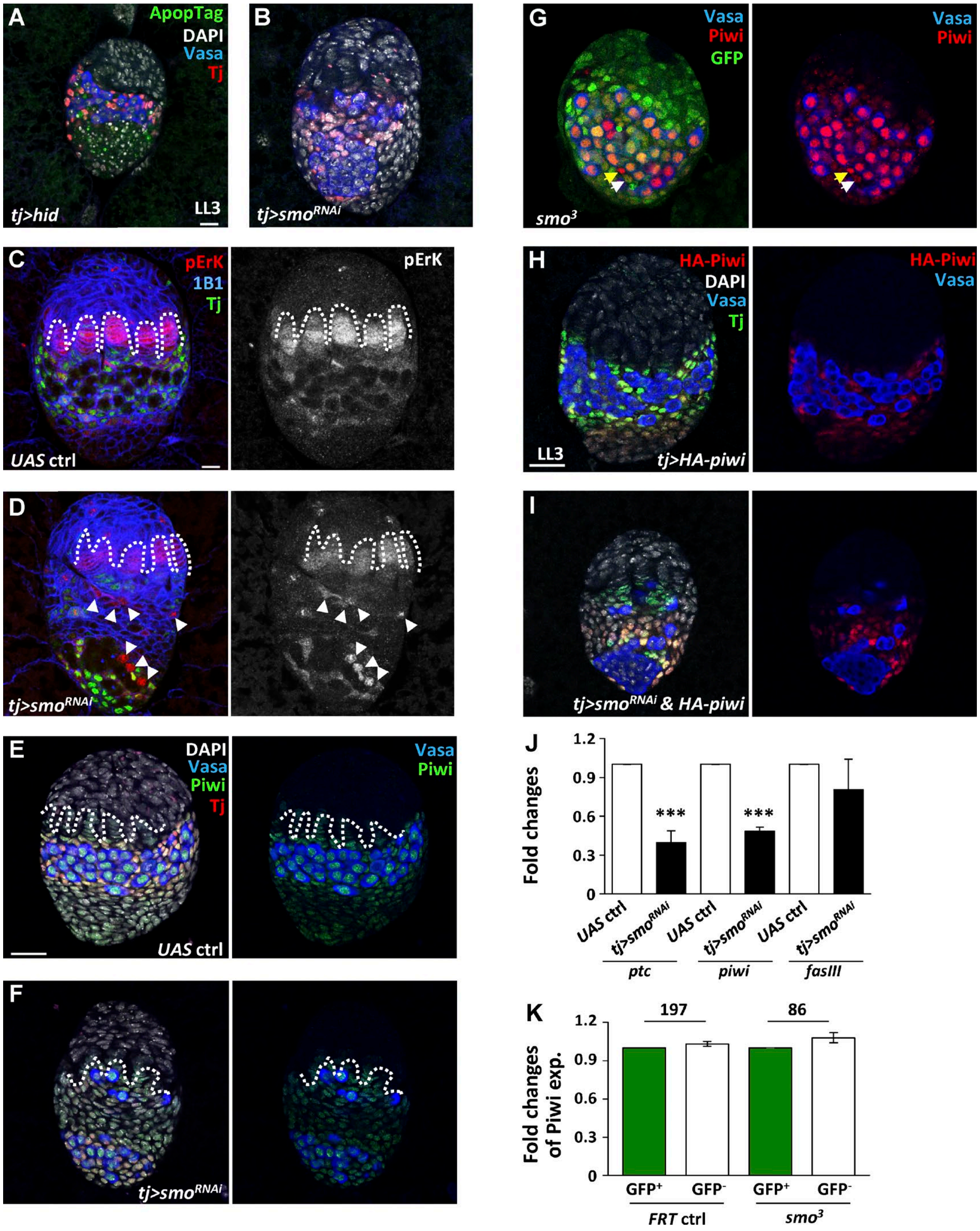

Figure S4. **Suppression of somatic Hh signaling does not induce cell death, decrease Egfr signaling, or decrease Piwi expression.** (A and B) Knockdown somatic smo does not induce cell death in larval gonads. Late-L3 *tj>hid* (positive control; A) and *tj>smo*^{RNAi} gonads (B) with ApopTag (green, dead cells), Tj (red, ICs), Vasa (blue, PGCs), and DAPI (gray, DNA). ApopTag signals overlapping with fragmented DNA are abundantly detected in the gonads overexpressing the proapoptotic gene, *hid*, driven by *tj-GAL4*, but rarely detected in *smo*-knockdown gonads. (C and D) Knockdown somatic smo does not reduce Egfr signaling in larval gonads. Late-L3 control (ctrl; C) and *tj>smo*^{RNAi} (D) gonads with phospho (p)-Erk (red in C, left, and D, left; gray in C, right, and D, right), Tj (green), and 1B1 (blue, PGC fusomes and somatic cell membranes). (E–K) Piwi is dispensable for soma-germline interaction controlled by Hh signaling. (E and F) Late-L3 control (ctrl; E) and *tj>smo*^{RNAi} (F) with Piwi (green), Tj (red, ICs), Vasa (blue, PGCs), and DAPI (gray, DNA). (G) Late-L3 *smo*^{3} mutant mosaic gonads with GFP (green, wild-type cells), Vasa (blue), and Piwi (red). White arrows indicate GFP^{+} wild-type ICs, and yellow arrows indicate GFP^{-} control and *smo*^{3} mutant ICs. (H and I) Late-L3 control (H) and *tj>smo*^{RNAi} *& HA-piwi* (I) with HA (red), Tj (green), Vasa (blue), and DAPI (gray). Dashed lines mark forming terminal filaments. Bars, 10 µm. (J) Mean fold changes of *ptc*, *piwi*, and *fasIII* transcript levels in *tj>smo*^{RNAi} gonads relative to that in control gonads. (K) Mean fold changes of Piwi expression (exp.) in GFP^{-} ICs relative to those of neighboring GFP^{+} wild-type ICs of late-L3 mock and *smo*^{3} mutant mosaic gonads. Number of ICs analyzed is shown above each bar. Statistical differences were analyzed by two-tailed *t* test. Error bars represent SEM. *, $P < 0.05$; ***, $P < 0.001$.

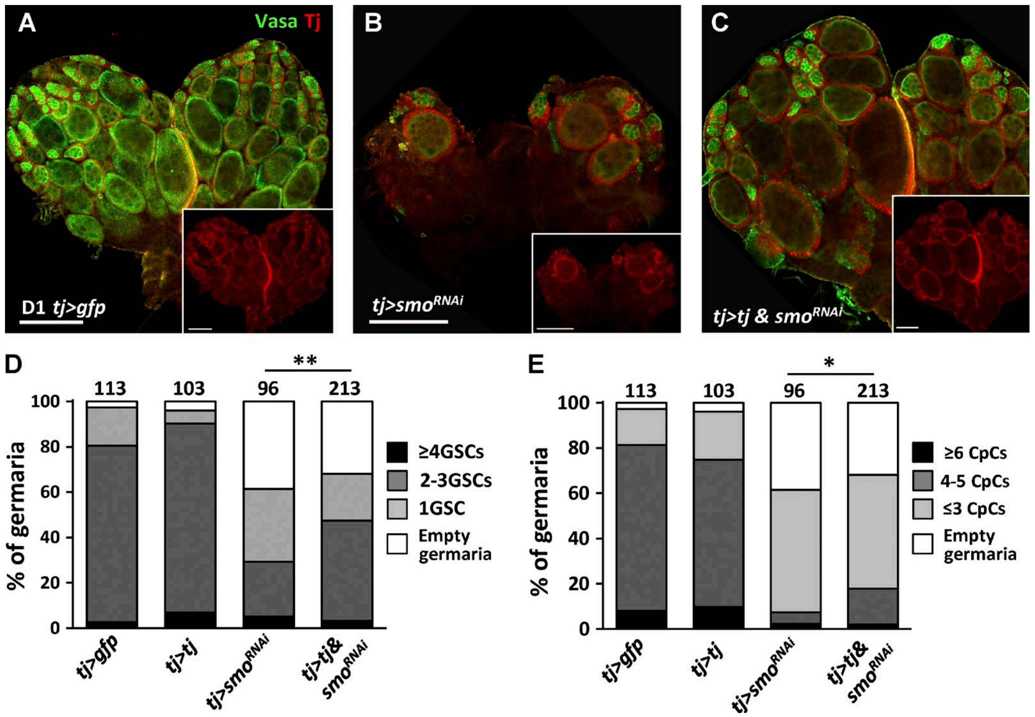

Figure S5. **Overexpression of *tj* in *smo*-knockdown gonads partially rescues ovary morphogenesis, niche cap cells, and GSC numbers.** (A–C) Newly eclosed (D1) *tj>gfp* (A), *tj>smo*^{RNAi} (B), and *tj>tj & smo*^{RNAi} (C) with Tj (red; cap, escort, and follicle cell nuclei) and Vasa (green, germ cells). Bars, 50 µm. (D and E) GSC (D) and cap cell (E) numbers in germaria of *tj>gfp*, *tj>smo*^{RNAi}, and *tj>tj & smo*^{RNAi} newly eclosed ovaries. Numbers of germaria analyzed are shown above each bar. Statistical differences were analyzed by χ^2. Error bars represent SEM. ***, $P < 0.001$.
